# Supplementary figures and images for: Fatal human H3N8 influenza virus has a moderate pandemic risk
Source: PLoS Pathog. 2026 Mar 3;22(3):e1013586. doi: 10.1371/journal.ppat.1013586 (PMC12970972; doi:10.1371/journal.ppat.1013586)

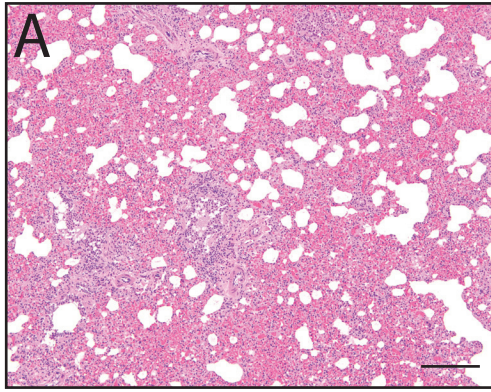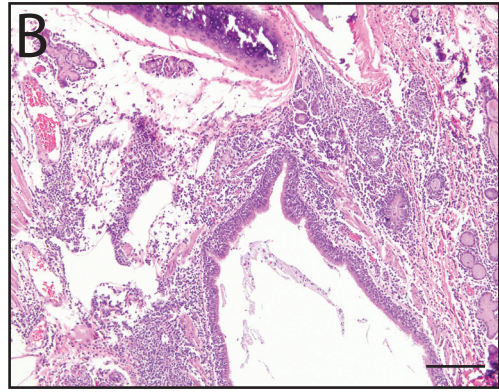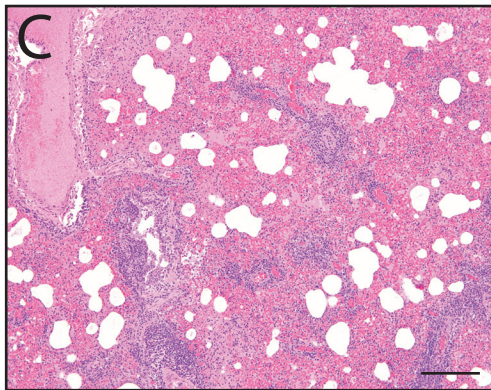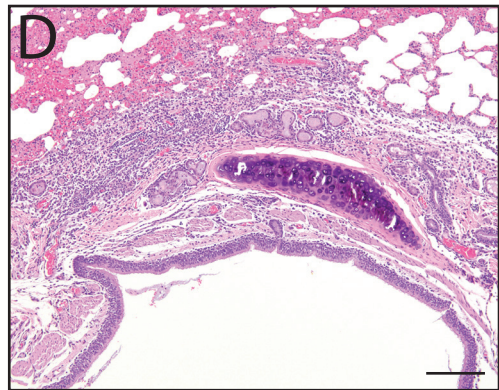

Supplement: S1 Fig — Representative lung images from A/GD/F005/23 H3N8 infected ferrets at day 5 post-infection. A, B are from ferrets without pre-existing immunity and C, D are from ferrets with H3N2 pre-existing immunity. Scale bar 200 μm. (PDF) [file ppat.1013586.s001.pdf]
