# Supplementary material for: Fatal human H3N8 influenza virus has a moderate pandemic risk
Source: PLoS Pathog. 2026 Mar 3;22(3):e1013586. doi: 10.1371/journal.ppat.1013586 (PMC12970972; doi:10.1371/journal.ppat.1013586)
Supplement: S1 Table — The table compares the amino acid differences between each pair of isolates, where each row represents one human isolate. Each cell represents the pairwise comparison between that isolate and the isolate in the corresponding column. The identities of each amino acid represent the amino acid encoded for the isolate in each row. For example, A/Henan differs from A/Guangdong at 2 amino acid sites in NP: 50 and 55. A/Henan encodes a C at 50 and an F at 55, while A/Guangdong encodes an F at 50 and an I at 55. (DOCX) [file ppat.1013586.s002.docx]

**S1 Table. A pairwise comparison of the three human H3N8 samples show they differ by multiple nonsynonymous amino acid (AA) substitutions across their genomes.** The table compares the amino acid differences between each pair of isolates, where each row represents one human isolate. Each cell represents the pairwise comparison between that isolate and the isolate in the corresponding column. The identities of each amino acid represent the amino acid encoded for the isolate in each row. For example, A/Henan differs from A/Guangdong at 2 amino acid sites in MP: 50 and 55. A/Henan encodes a C at 50 and an F at 55, while A/Guangdong encodes an F at 50 and an I at 55.
