# Supplementary material for: Fatal human H3N8 influenza virus has a moderate pandemic risk
Source: PLoS Pathog. 2026 Mar 3;22(3):e1013586. doi: 10.1371/journal.ppat.1013586 (PMC12970972; doi:10.1371/journal.ppat.1013586)
Supplement: S2 Table — (PDF) [file ppat.1013586.s003.pdf]

Table S2: Accession Numbers

Strain	GISAID_Accessi	Genbank_Accessi	Submitting Lab	Originating Lab
a/duck/france/161005/2016		MH560271	French Agency for Food, Environmental and Occupational Health & Sa	unknown
a/ruddy_turnstone/new_jersey/260/1990		CY101126	on behalf of JCVI/St. Jude Children's Research Hospital/NCBI, Nation	unknown
a/semi-palmated_sandpiper/new_jersey/253/1990		CY101635	on behalf of JCVI/St. Jude Children's Research Hospital/NCBI, Nation	unknown
a/laughing_gull/new_jersey/180/1990		CY101627	on behalf of JCVI/St. Jude Children's Research Hospital/NCBI, Nation	unknown
a/semi-palmated_sandpiper/new_jersey/237/1990		CY101118	on behalf of JCVI/St. Jude Children's Research Hospital/NCBI, Nation	unknown
a/ruddy_turnstone/new_jersey/138/1990		CY101105	on behalf of JCVI/St. Jude Children's Research Hospital/NCBI, Nation	unknown
a/herring_gull/new_jersey/159/1990		CY101619	on behalf of JCVI/St. Jude Children's Research Hospital/NCBI, Nation	unknown
a/mallard_duck/netherlands/3/2001	EPI1306877		unknown	unknown
a/common_teal/netherlands/07/2000	EPI1013727		unknown	unknown
a/common_teal/netherlands/7/2000	EPI485544		unknown	unknown
a/mallard_duck/netherlands/08/2000	EPI1013399		unknown	unknown
a/mallard/netherlands/2/1999	EPI251661		unknown	unknown
a/mallard_duck/netherlands/34/2006	EPI890870		unknown	unknown
a/bantam/nanchang/9-366/2000	EPI90334		unknown	unknown
a/pigeon/nanchang/9-058/2000	EPI90332		unknown	unknown
a/duck/thailand/ay-354/2008		FJ802401	Chulalongkorn University	unknown
a/duck/hebei/b1646-2/2011	EPI774955		unknown	unknown
a/duck/hebei/b1645-2/2011		KU158891	Chinese Academy of Agricultural Sciences, Shanghai Veterinary Resea	unknown
a/duck/hebei/b1647-1/2011		KU158893	Chinese Academy of Agricultural Sciences, Shanghai Veterinary Resea	unknown
a/aquatic_bird/korea/kn-4/2005		EU301219	Chungbuk National University, College of Medicine and Medical Rese	unknown
a/aquatic_bird/korea/jn-1/2005		EU301214	Chungbuk National University, College of Medicine and Medical Rese	unknown
a/aquatic_bird/korea/kn-2/2005	EPI126969		unknown	unknown
a/aquatic_bird/korea/kn-1/2004		EU301216	Chungbuk National University, College of Medicine and Medical Rese	unknown
a/aquatic_bird/korea/kn-3/2005		EU301218	Chungbuk National University, College of Medicine and Medical Rese	unknown
a/turkey/england/1969		AY531037	Statens Serum Institut, Department of Virology	unknown
a/turkey/england/69	EPI242693		unknown	unknown
a/duck/ukraine/1/1963	EPI317610		Friedrich-Loeffler-Institut	Friedrich-Loeffler-Institut
a/duck/ukraine/1963	EPI407884		unknown	unknown
a/swine/fujian/668/01		AY857957	Gansu Agriculture University, Veterinary Medicine Department	unknown
a/swine/inner_mongolia/547/2001		DQ021910	Harbin Veterinary Research Institute, National Key Laboratory of Biot	unknown
a/mallard/iceland/1007/2011	EPI475925		unknown	unknown
a/mallard/netherlands/3/2005		CY041242	on behalf of JCVI/Erasmus Medical College/NCBI, National Center for	unknown
a/budgerigar/aichi/1/77	EPI1120588		National Institute of Animal Health	National Institute of Animal Health
a/swine/kazakhstan/106/1985	EPI558651		unknown	unknown
a/chicken/pakistan/narc-16945/2010		HQ165996	Animal Sciences Institute, National Agricultural Research Centre, Na	unknown
a/mallard/italy/4394-10/05	EPI301863		Istituto Zooprofilattico Sperimentale Delle Venezie	Istituto Zooprofilattico Sperimentale Delle Venezie
a/mallard/sweden/50/2002		CY060308	National Influenza Center and Department of Virology, Erasmus Medic	unknown
a/duck/norway/1/03	EPI13713		unknown	unknown
a/duck/norway/1/2003		AJ841293	Jonassen C.M., Section of Virology and Serology, National Veterinary I	unknown
a/wild_duck/germany/wv2555/2006	EPI185349		Friedrich-Loeffler-Institut	unknown
a/duck/germany/r2555/2006	EPI1841254		Friedrich-Loeffler-Institut	Chemisches und Veterin√§runtersuchungsamt Stut
a/stellers_eider/alaska/44222-192/2006	EPI328758		unknown	unknown
a/pygoscelis_papua/fildes_peninsula__king_george_island/31/2023	EPI3349814		Central Research Institute of Epidemiology	Research Institute of Experimental and Clinical Me
a/duck/south_africa/1108/2004		EF041487	ARC-Onderstepoort Veterinary Institute, Biotechnology	unknown
a/pelican/zambia/01/2006	EPI225337		unknown	unknown
a/mallard/hungary/19616/2007		GQ240821	National Veterinary Institute, R&D Section, Dept. of Virology, Unit of V	unknown
a/duck/moscow/3554/2008		GU991376	The Chumakov Institute of Poliomyelitis and Viral Encephalitis, The Ru	unknown
a/duck/moscow/3556/2008		MN692228	Chumakov Federal Scientific Center for Research and Development c	unknown

a/mallard/czech_republic/13577-24k/2010	EPI316760		unknown	unknown
a/anas_platyrhynchos/belgium/3950-8/2015	EPI1774448		unknown	unknown
a/mallard_duck/netherlands/56/2015	EPI1306910		unknown	unknown
a/common_teal/georgia/1/2011		KC541684	Erasmus Medical Center, Departament of Virology and National Influe	unknown
a/common_teal/republic_of_georgia/1/2011	EPI618046		unknown	unknown
a/duck/moscow/4780/2012		MT773363	The Gamaleya National Research Centre for Epidemiology and Microb	unknown
a/duck/moscow/4788/2012		MT773386	The Gamaleya National Research Centre for Epidemiology and Microb	unknown
a/mallard/65112/03	EPI235184		unknown	unknown
a/turnstone/netherlands/3/2008	EPI890320		unknown	unknown
a/ruddy_turnstone/netherlands/6/2008	EPI1307459		unknown	unknown
a/turnstone/netherlands/4/2008	EPI890316		unknown	unknown
a/duck/nanchang/8-174/2000	EPI90340		unknown	unknown
a/turnstone/netherlands/1/2010	EPI890779		unknown	unknown
a/mule_duck/bulgaria/248/2009		KP714429	Infectious Diseases, St Jude Children's Hospital	unknown
a/mule_duck/bulgaria/101/2010		KP714442	Infectious Diseases, St Jude Children's Hospital	unknown
a/mule_duck/bulgaria/88/2010		KP714409	Infectious Diseases, St Jude Children's Hospital	unknown
a/mallard/czech_republic/14516/2007		JF682614	State Veterinary Institute Prague, National Reference Laboratory for A	unknown
a/mallard/norway/10_779/2009	EPI398946		unknown	unknown
a/mallard/sweden/101487/2009	EPI845220		unknown	unknown
a/mallard/georgia/3/2011		KC541685	Erasmus Medical Center, Departament of Virology and National Influe	unknown
a/mallard/republic_of_georgia/3/2011	EPI617976		unknown	unknown
a/duck/moscow/4203/2010	EPI1785246		unknown	unknown
a/duck/moscow/4238/2010	EPI1785254		unknown	unknown
a/duck/moscow/4298/2010		MN700159	The Gamaleya National Research Centre for Epidemiology and Microb	unknown
a/mallard/germany/439/2004	EPI859643		Robert Koch Institute Nationales Referenzzentrum f√ºr Influenza	Friedrich-Loeffler-Institut
a/mallard/germany/wv437/2004	EPI185346		Friedrich-Loeffler-Institut	unknown
a/mallard/germany-bw/sr632/2008	EPI279922		Friedrich-Loeffler-Institut	unknown
a/mallard/germany-bw/sr530/2007	EPI279921		Friedrich-Loeffler-Institut	unknown
a/mallard/germany-bw/sr520/2007	EPI279920		Friedrich-Loeffler-Institut	unknown
a/mallard/germany-bw/sr519/2007	EPI279919		Friedrich-Loeffler-Institut	unknown
a/mallard/finland/11237/2007		KF183636	Haartman Institute, University of Helsinki, Department of Virology	unknown
a/mallard/finland/12072/06		EU493448	Haartman Institute, University of Helsinki, Department of Virology	unknown
a/mallard_duck/netherlands/1/2004	EPI1011141		unknown	unknown
a/mallard_duck/netherlands/1/2003	EPI1010651		unknown	unknown
a/mallard_duck/netherlands/2/2003	EPI1013536		unknown	unknown
a/common_teal/sweden/1/2003	EPI251612		unknown	unknown
a/mallard/netherlands/5/2001	EPI251669		unknown	unknown
a/mallard/switzerland/wv4060167/2006		GQ415320	Institute of Virology and Immunoprophylaxis, Development	unknown
a/wigeon/italy/3818-34/05	EPI301864		Istituto Zooprofilattico Sperimentale Delle Venezie	Istituto Zooprofilattico Sperimentale Delle Venezie
a/bewick_swan/netherlands/5/2007	EPI1013744		unknown	unknown
a/mallard/sweden/1636/2002	EPI617138		unknown	unknown
a/mallard_duck/netherlands/13/2012		KX979517	Center for Research on Influenza Pathogenesis (CRIP)	unknown
a/anas_platyrhynchos/belgium/8295/2016	EPI1774528		unknown	unknown
a/arenaria_interpres/belgium/9365_2/2019		MT407145	Istituto Zooprofilattico Sperimentale delle Venezie, EU/OIE Reference	unknown
a/duck/moscow/5172/2015		OP133390	The Gamaleya National Research Centre for Epidemiology and Microb	unknown
a/duck/moscow/5163/2015		OP132936	The Gamaleya National Research Centre for Epidemiology and Microb	unknown
a/duck/moscow/5171/2015		OP133380	The Gamaleya National Research Centre for Epidemiology and Microb	unknown
a/duck/moscow/5169/2015		OP133013	The Gamaleya National Research Centre for Epidemiology and Microb	unknown
a/mallard/germany/r529/2008	EPI185336		Friedrich-Loeffler-Institut	unknown
a/mallard/italy/3342/2006	EPI554982		Istituto Zooprofilattico Sperimentale Delle Venezie	Istituto Zooprofilattico Sperimentale Delle Venezie
a/mallard/germany/r752/2008	EPI185338		Friedrich-Loeffler-Institut	unknown

a/turnstone/netherlands/1/2007	EPI1537034		unknown	unknown
a/dunlin/netherlands/3/2007	EPI890998		unknown	unknown
a/turnstone/netherlands/2/2007	EPI890799		unknown	unknown
a/dunlin/netherlands/2/2007	EPI890193		unknown	unknown
a/mallard/netherlands/11013808/2011	EPI1140121		Wageningen Bioveterinary Research	Wageningen Bioveterinary Research
a/eurasian_teal/netherlands/1/2011	EPI891113		unknown	unknown
a/mallard/czech_republic/14333-1k/2011		JQ737213	State Veterinary Institute Prague, National Reference Laboratory for A	unknown
a/mallard/sweden/140959/2013		KT725408	Linnaeus University, Department of Health and Life Science	unknown
a/mallard/sweden/141342/2013		KT725411	Linnaeus University, Department of Health and Life Science	unknown
a/mallard/sweden/141675/2013		KT725420	Linnaeus University, Department of Health and Life Science	unknown
a/mallard/sweden/141462/2013		KT725414	Linnaeus University, Department of Health and Life Science	unknown
a/mallard/sweden/141811/2013		KT725426	Linnaeus University, Department of Health and Life Science	unknown
a/mallard/sweden/140901/2013		KT725402	Linnaeus University, Department of Health and Life Science	unknown
a/mallard/sweden/140958/2013		KT725405	Linnaeus University, Department of Health and Life Science	unknown
a/mallard/sweden/141711/2013		KT725423	Linnaeus University, Department of Health and Life Science	unknown
a/common_eider/netherlands/1/2006		CY041338	on behalf of JCVI/Erasmus Medical College/NCBI, National Center for	unknown
a/mallard/germany-be/r663/08	EPI416239		Friedrich-Loeffler-Institut	unknown
a/mallard/germany-be/r663/2008	EPI279928		Friedrich-Loeffler-Institut	unknown
a/duck/zambia/04/2008	EPI310341		unknown	unknown
a/goose/zambia/05/2008	EPI310349		unknown	unknown
a/goose/zambia/06/2008	EPI310357		unknown	unknown
a/mallard/germany-he/r1648/2007	EPI279926		Friedrich-Loeffler-Institut	unknown
a/anas_plathyrhynchos/spain/0454/2006		FN386461	Nunez J.I., Cresa, departamento de infecciones viricas, edifici Cresa,	unknown
a/duck/italy/3139-2/06	EPI301854		Istituto Zooprofilattico Sperimentale Delle Venezie	Istituto Zooprofilattico Sperimentale Delle Venezie
a/duck/italy/194659/2006		FJ432762	Istituto Zooprofilattico Sperimentale della Lombardia e dell' Emilia Ro	unknown
a/mallard/netherlands/39/2008	EPI1537086		unknown	unknown
a/mallard_duck/netherlands/51/2008	EPI1013674		unknown	unknown
a/mallard_duck/netherlands/14/2011	EPI1010590		unknown	unknown
a/mallard_duck/netherlands/17/2011	EPI889691		unknown	unknown
a/mallard/netherlands/28/2006		CY076905	on behalf of JCVI/Erasmus Medical College/NCBI, National Center for	unknown
a/mallard/netherlands/1/2007		CY043816	on behalf of JCVI/Erasmus Medical College/NCBI, National Center for	unknown
a/anas_platyrhynchos/belgium/12827/2007		HM570058	Veterinary and Agrochemical Research Institute	unknown
a/duck/netherlands/06027358/2006	EPI1230087		Wageningen Bioveterinary Research	Wageningen Bioveterinary Research
a/gadwall_duck/netherlands/3/2006	EPI890484		unknown	unknown
a/mallard_duck/netherlands/60/2006	EPI1014560		unknown	unknown
a/mallard/ukraine/05842-namru3/2006	EPI372504		Centers for Disease Control and Prevention	U.S. Naval Medical Research Unit No.3
a/mallard/netherlands/21/2013	EPI1530584		unknown	unknown
a/mallard_duck/netherlands/16/2012	EPI1010831		unknown	unknown
a/mallard_duck/netherlands/30/2010	EPI889752		unknown	unknown
a/mallard_duck/netherlands/13/2009	EPI1010971		unknown	unknown
a/mallard_duck/netherlands/62/2008	EPI1307513		unknown	unknown
a/mallard/germany-rp/r2322/07	EPI279927		Friedrich-Loeffler-Institut	unknown
a/duck/italy/6207/08	EPI301853		Istituto Zooprofilattico Sperimentale Delle Venezie	Istituto Zooprofilattico Sperimentale Delle Venezie
a/mallard/germany-bw/sr871/2008	EPI279924		Friedrich-Loeffler-Institut	unknown
a/mallard/germany-bw/sr872/2008	EPI279925		Friedrich-Loeffler-Institut	unknown
a/chicken/italy/3582-51/10	EPI301855		Istituto Zooprofilattico Sperimentale Delle Venezie	Istituto Zooprofilattico Sperimentale Delle Venezie
a/mallard/netherlands/3/2013	EPI1530608		unknown	unknown
a/mallard_duck/netherlands/2/2013	EPI1014857		unknown	unknown
a/mallard_duck/netherlands/6/2008	EPI1307236		unknown	unknown
a/mallard_duck/netherlands/7/2008	EPI889787		unknown	unknown
a/duck/moscow/4242/2010		MN700151	The Gamaleya National Research Centre for Epidemiology and Microb	unknown

a/mallard/netherlands/14016619/2014	EPI1140209		Wageningen Bioveterinary Research	Wageningen Bioveterinary Research
a/mallard_duck/netherlands/6/2013	EPI891013		unknown	unknown
a/mallard/netherlands/11015220/2011	EPI1140129		Wageningen Bioveterinary Research	Wageningen Bioveterinary Research
a/duck/moscow/4661/2011	EPI1785302		unknown	unknown
a/barnacle_goose/netherlands/2/2014	EPI1010875		unknown	unknown
a/grey_seal/england/027661/2017	EPI1566822		Animal and Plant Health Agency (APHA)	Animal and Plant Health Agency (APHA)
a/duck/vietnam/lbm48/2011	EPI574306		unknown	unknown
a/garganey/korgalzhyn/865/2004		GU953258	Institute of Microbiology and Virology, Laboratory for Viral Ecology	unknown
a/white-fronted_goose/mongolia/1-125/2008		JN029678	National Veterinary Research and Quarantine Service, Avian Disease	unknown
a/red-crested_pochard/mongolia/463v/2009		KF501090	School of Public Health, The University of Hong Kong	unknown
a/snow_goose/mongolia/1-29/2008		JN029662	National Veterinary Research and Quarantine Service, Avian Disease	unknown
a/snow_goose/mongolia/1-44/2008		JN029670	National Veterinary Research and Quarantine Service, Avian Disease	unknown
a/mallard_duck/netherlands/34/2010	EPI1010947		unknown	unknown
a/duck/moscow/4494/2011		MN759699	The Gamaleya National Research Centre for Epidemiology and Microb	unknown
a/duck/moscow/4681/2011		MN759707	The Gamaleya National Research Centre for Epidemiology and Microb	unknown
a/pintail/egypt/mb-d-384c/2015		MN210316	St. Jude Center of Excellence for Influenza Research and Surveillance	unknown
a/duck/bangladesh/26948/2015	EPI965377		unknown	unknown
a/duck/bangladesh/26974/2015	EPI965287		unknown	unknown
a/duck/bangladesh/26920/2015	EPI965495		unknown	unknown
a/duck/bangladesh/26918/2015	EPI965193		unknown	unknown
a/duck/bangladesh/35669/2018	EPI1581578		unknown	unknown
a/pintail/novosibirsk_region/506k/2018	EPI1353858		National Institute of Animal Health	Research Institute of Experimental and Clinical Me
a/common_teal/chany_lake/357/2019	EPI1638420		National Institute of Animal Health	Research Institute of Experimental and Clinical Me
a/mallard/omsk_region/46/2020	EPI1847623		National Institute of Animal Health	Research Institute of Experimental and Clinical Me
a/garganey/omsk_region/22/2019	EPI1638556		National Institute of Animal Health	Research Institute of Experimental and Clinical Me
a/shoveler/omsk_region/71/2019	EPI1638588		National Institute of Animal Health	Research Institute of Experimental and Clinical Me
a/mallard/novosibirsk_region/963k/2018	EPI1353866		National Institute of Animal Health	Research Institute of Experimental and Clinical Me
a/duck/bangladesh/18d1577/2021	EPI1985988		International Centre for Diarrhoeal Disease Research (ICDDR,B)	icddr,b International Centre for Diarrhoeal Disease
a/duck/bangladesh/18d1578/2021	EPI1985995		International Centre for Diarrhoeal Disease Research (ICDDR,B)	icddr,b International Centre for Diarrhoeal Disease
a/mallard/omsk_region/63/2019	EPI1638476		National Institute of Animal Health	Research Institute of Experimental and Clinical Me
a/duck/mongolia/876/2019		MT020270	Hokkaido University, Faculty of Veterinary Medicine	unknown
a/duck/mongolia/961/2019	EPI1777597		unknown	unknown
a/tufted_duck/novosibirsk_region/989k/2018	EPI1355579		National Institute of Animal Health	Research Institute of Experimental and Clinical Me
a/gadwall/chany/893/2018	EPI1333763		State Research Center of Virology and Biotechnology (VECTOR)	State Research Center of Virology and Biotechnolog
a/duck/bangladesh/19-d-1955/2022	EPI3115913		Centers for Disease Control and Prevention	icddr,b International Centre for Diarrhoeal Disease
a/duck/bangladesh/19d2073/2023	EPI3116013		unknown	unknown
a/mallard/novosibirsk_region/957k/2018	EPI1352470		National Institute of Animal Health	Research Institute of Experimental and Clinical Me
a/wild_duck/shandong/w11397/2019	EPI2245888		Liaocheng University	College of Agronomy, Liaocheng University
a/anser_fabalis/china/d338/2020	EPI2181588		unknown	unknown
a/wild_duck/south_korea/knu2020-104/2020		OK236003	Zoonosis Research Center, School of Medicine, Department of Infecti	unknown
a/eurasian_wigeon/south_korea/knu2020-83/2020		PP746164	Wonkwang University School of Medicine, Infection Biology	unknown
a/goose/anhui/ah0785/2021		OM527229	Yangzhou University, College of Veterinary Medicine	unknown
a/northern_shoveler/north-kazakhstan/20/2018	EPI1790215		unknown	unknown
a/duck/bangladesh/46090/2020	EPI2147613		unknown	unknown
a/duck/mongolia/2021-mg02/2021		OP692510	Wonkwang University School of Medicine, Department of Infection Bi	unknown
a/duck/mongolia/mn18-14/2018		MW487352	Zoonosis Research Center, School of Medicine, Department of Infecti	unknown
a/wild_duck/shandong/w6280/2019	EPI2245864		Liaocheng University	College of Agronomy, Liaocheng University
a/mallard_anas_platyrhynchos_/south_korea/knu2021-17/2021	EPI2153471		unknown	unknown
a/wild_duck/shandong/w6284/2019	EPI2245872		Liaocheng University	College of Agronomy, Liaocheng University
a/duck/mongolia/914/2018	EPI1510694		unknown	unknown
a/duck/mongolia/921/2018		MK979160	Hokkaido University, Faculty of Veterinary Medicine	unknown

a/duck/mongolia/920/2018		MK979152	Hokkaido University, Faculty of Veterinary Medicine	unknown
a/duck/mongolia/915/2018	EPI1510702		unknown	unknown
a/duck/mongolia/620/2018		MK978976	Hokkaido University, Faculty of Veterinary Medicine	unknown
a/duck/mongolia/723/2018	EPI1510606		unknown	unknown
a/duck/mongolia/726/2018	EPI1510614		unknown	unknown
a/duck/mongolia/618/2018	EPI1510574		unknown	unknown
a/duck/mongolia/919/2018	EPI1510734		unknown	unknown
a/duck/mongolia/917/2018		MK979128	Hokkaido University, Faculty of Veterinary Medicine	unknown
a/duck/mongolia/916/2018	EPI1510710		unknown	unknown
a/duck/mongolia/906/2018		MK979080	Hokkaido University, Faculty of Veterinary Medicine	unknown
a/duck/mongolia/918/2018		MK979136	Hokkaido University, Faculty of Veterinary Medicine	unknown
a/duck/mongolia/941/2018	EPI1510782		unknown	unknown
a/duck/mongolia/972/2018		MK979232	Hokkaido University, Faculty of Veterinary Medicine	unknown
a/duck/mongolia/922/2018	EPI1510758		unknown	unknown
a/shoveler/chany/82k/2014	EPI884239		Research Institute of Experimental and Clinical Medicine	Research Institute of Experimental and Clinical Me
a/common_shelduck/mongolia/2076/2011		KF501077	School of Public Health, The University of Hong Kong	unknown
a/duck/mongolia/30/2015		LC121236	Graduate School of Veterinary Medicine; Kita-18 Nishi-9, Kita-ku, Sap	unknown
a/duck/moscow/5037/2014		MT773423	The Gamaleya National Research Centre for Epidemiology and Microb	unknown
a/anas_platyrhynchos/belgium/7827/2018	EPI1774496		unknown	unknown
a/mallard/netherlands/22014777-004/2022	EPI2178884		Wageningen Bioveterinary Research	Wageningen Bioveterinary Research
a/anas_platyrhynchos/belgium/827/2020	EPI1942357		Sciensano, Department of Animal Infectious Diseases	Sciensano - Animal Infectious Diseases
a/teal/dagestan/44d/2018	EPI1353408		National Institute of Animal Health	Research Institute of Experimental and Clinical Me
a/mallard/netherlands/21/2016	EPI1537045		unknown	unknown
a/mallard/netherlands/37/2015	EPI1530593		unknown	unknown
a/barnacle_goose/malaysia/vri-6728/2018	EPI2695511		unknown	unknown
a/paradise_shelduck/malaysia/vri-6731/2018	EPI2695519		unknown	unknown
a/gallus_gallus/belgium/4464/2019	EPI1788765		unknown	unknown
a/gallus_gallus/belgium/7200_0001/2019__h3n1_	EPI1889063		Sciensano, Department of Animal Infectious Diseases	Sciensano - Animal Infectious Diseases
a/gallus_gallus/belgium/6519/2019	EPI1788774		unknown	unknown
a/gallus_gallus/belgium/5826_0002/2019_h3n1_	EPI1888963		Sciensano, Department of Animal Infectious Diseases	Sciensano - Animal Infectious Diseases
a/gallus_gallus/belgium/6121_0002/2019_h3n1_	EPI1888971		Sciensano, Department of Animal Infectious Diseases	Sciensano - Animal Infectious Diseases
a/gallus_gallus/belgium/6079/2019	EPI1788772		unknown	unknown
a/gallus_gallus/belgium/4753_0001/2019_h3n1_	EPI1888708		Sciensano, Department of Animal Infectious Diseases	Sciensano - Animal Infectious Diseases
a/gallus_gallus/belgium/5113_0001/2019_h3n1_	EPI1888827		Sciensano, Department of Animal Infectious Diseases	Sciensano - Animal Infectious Diseases
a/gallus_gallus/belgium/5055_0003/2019_h3n1_	EPI1888679		Sciensano, Department of Animal Infectious Diseases	Sciensano - Animal Infectious Diseases
a/gallus_gallus/belgium/6986/2019_h3n1_	EPI1889071		Sciensano, Department of Animal Infectious Diseases	Sciensano - Animal Infectious Diseases
a/gallus_gallus/belgium/5597/2019	EPI1788769		unknown	unknown
a/gallus_gallus/belgium/5766_0001/2019_h3n1_	EPI1888947		Sciensano, Department of Animal Infectious Diseases	Sciensano - Animal Infectious Diseases
a/gallus_gallus/belgium/4492_0002/2019_h3n1_	EPI1888639		Sciensano, Department of Animal Infectious Diseases	Sciensano - Animal Infectious Diseases
a/gallus_gallus/belgium/4846_0002/2019_h3n1_	EPI1888761		Sciensano, Department of Animal Infectious Diseases	Sciensano - Animal Infectious Diseases
a/gallus_gallus/belgium/4008/2019_h3n1_	EPI1888403		Sciensano, Department of Animal Infectious Diseases	Sciensano - Animal Infectious Diseases
a/gallus_gallus/belgium/3953/2019_h3n1_	EPI1888377		Sciensano, Department of Animal Infectious Diseases	Sciensano - Animal Infectious Diseases
a/gallus_gallus/belgium/4327_0001/2019_h3n1_	EPI1888435		Sciensano, Department of Animal Infectious Diseases	Sciensano - Animal Infectious Diseases
a/gallus_gallus/belgium/4890_0002/2019_h3n1_	EPI1888777		Sciensano, Department of Animal Infectious Diseases	Sciensano - Animal Infectious Diseases
a/gallus_gallus/belgium/4849_0001/2019__h3n1_	EPI1888443		Sciensano, Department of Animal Infectious Diseases	Sciensano - Animal Infectious Diseases
a/gallus_gallus/belgium/5228_0001/2019__h3n1_	EPI1888595		Sciensano, Department of Animal Infectious Diseases	Sciensano - Animal Infectious Diseases
a/gallus_gallus/belgium/6986/2019	EPI1788775		unknown	unknown
a/gallus_gallus/belgium/4010/2019_h3n1_	EPI1888395		Sciensano, Department of Animal Infectious Diseases	Sciensano - Animal Infectious Diseases
a/gallus_gallus/belgium/4428_0001/2019__h3n1_	EPI1888531		Sciensano, Department of Animal Infectious Diseases	Sciensano - Animal Infectious Diseases
a/gallus_gallus/belgium/4464_0001/2019__h3n1_	EPI1888539		Sciensano, Department of Animal Infectious Diseases	Sciensano - Animal Infectious Diseases
a/gallus_gallus/belgium/4491_0006/2019__h3n1_	EPI1888718		Sciensano, Department of Animal Infectious Diseases	Sciensano - Animal Infectious Diseases

a/gallus_gallus/belgium/4581_0002/2019_h3n1_	EPI1888419		Sciensano, Department of Animal Infectious Diseases	Sciensano - Animal Infectious Diseases
a/gallus_gallus/belgium/4768_0002/2019_h3n1_	EPI1888742		Sciensano, Department of Animal Infectious Diseases	Sciensano - Animal Infectious Diseases
a/gallus_gallus/belgium/5534_0001/2019_h3n1_	EPI1888894		Sciensano, Department of Animal Infectious Diseases	Sciensano - Animal Infectious Diseases
a/gallus_gallus/belgium/5770_0001/2019_h3n1_	EPI1888941		Sciensano, Department of Animal Infectious Diseases	Sciensano - Animal Infectious Diseases
a/gallus_gallus/belgium/6082_0002/2019_h3n1_	EPI1889029		Sciensano, Department of Animal Infectious Diseases	Sciensano - Animal Infectious Diseases
a/garganey/chany_lake/20/2014	EPI1353400		National Institute of Animal Health	Research Institute of Experimental and Clinical Me
a/duck/mongolia/996/2015	EPI704446		unknown	unknown
a/duck/bangladesh/33137/2017	EPI1309677		unknown	unknown
a/duck/mongolia/521/2015		LC121404	Graduate School of Veterinary Medicine; Kita-18 Nishi-9, Kita-ku, Sap	unknown
a/duck/mongolia/199/2015		LC121316	Graduate School of Veterinary Medicine; Kita-18 Nishi-9, Kita-ku, Sap	unknown
a/duck/mongolia/208/2015		LC121332	Graduate School of Veterinary Medicine; Kita-18 Nishi-9, Kita-ku, Sap	unknown
a/duck/mongolia/777/2015		LC121460	Graduate School of Veterinary Medicine; Kita-18 Nishi-9, Kita-ku, Sap	unknown
a/duck/mongolia/83/2015	EPI693706		unknown	unknown
a/duck/mongolia/167/2015	EPI704315		unknown	unknown
a/duck/mongolia/211/2015	EPI704372		unknown	unknown
a/duck/bangladesh/19d705/2016	EPI1330324		Centers for Disease Control and Prevention	icddr,b International Centre for Diarrhoeal Disease
a/duck/bangladesh/17d747/2016	EPI1330318		Centers for Disease Control and Prevention	icddr,b International Centre for Diarrhoeal Disease
a/duck/bangladesh/41451/2019	EPI1903197		unknown	unknown
a/duck/mongolia/217/2018	EPI1818052		unknown	unknown
a/duck/bangladesh/34186/2017	EPI1555977		unknown	unknown
a/duck/bangladesh/34192/2017	EPI1555914		unknown	unknown
a/duck/bangladesh/34194/2017	EPI1556126		unknown	unknown
a/duck/bangladesh/34191/2017	EPI1556095		unknown	unknown
a/duck/bangladesh/34193/2017	EPI1556145		unknown	unknown
a/duck/jiangsu/j3784/2014		KP767637	China Animal Health and Epidemiology Center, Laboratory of Avian Di	unknown
a/duck/jiangsu/j3741/2014		KP767635	China Animal Health and Epidemiology Center, Laboratory of Avian Di	unknown
a/duck/jiangsu/j3722/2014		KP767634	China Animal Health and Epidemiology Center, Laboratory of Avian Di	unknown
a/duck/jiangsu/j3781/2014		KP767636	China Animal Health and Epidemiology Center, Laboratory of Avian Di	unknown
a/duck/jiangxi/01.14_ncjd060-p/2015_mixed_	EPI666237		Institute of Microbiology, Chinese Academy of Sciences	unknown
a/mallard/jiangxi/g98/2014	EPI1601911		unknown	unknown
a/swan_goose/jiangxi/h23/2014		MT375333	Tsinghua University, Department of earthsystem science	unknown
a/swan_goose/jiangxi/h23/2014_h3n6_	EPI1590544		Tsinghua University	Tsinghua University
a/swan_goose/jiangxi/h24/2014		MT375535	Tsinghua University, Department of earthsystem science	unknown
a/swan_goose/jiangxi/h24/2014_h3n6_	EPI1590552		Tsinghua University	Tsinghua University
a/duck/kerala/ducl1412100031/2014		MT272397	ICAR-National Institute of High Security Animal Disease, Diagnostic I	unknown
a/muscovy_duck/vietnam/lbm529/2013		AB916667	Avian Zoonosis Research Center, Faculty of Agriculture; 4-101 Koyam	unknown
a/duck/viet_nam/hn-1732/2014		MK964651	J. Craig Venter Institute	unknown
a/duck/viet_nam/hn-1734/2014		MK964659	J. Craig Venter Institute	unknown
a/duck/viet_nam/hn-1731/2014	EPI1580088		unknown	unknown
a/muscovy_duck/viet_nam/hn-1736/2014		MK964989	J. Craig Venter Institute	unknown
a/duck/viet_nam/hn-1730/2014	EPI1580080		unknown	unknown
a/muscovy_duck/viet_nam/hn-1740/2014		MK965021	J. Craig Venter Institute	unknown
a/duck/nha_trang/161/2016		LC496152	Avian Zoonosis Research Center, Faculty of Agriculture; 4-101, Koyam	unknown
a/duck/nha_trang/177/2016		LC496168	Avian Zoonosis Research Center, Faculty of Agriculture; 4-101, Koyam	unknown
a/duck/vietnam/hu8-1918/2017	EPI1557251		unknown	unknown
a/duck/vietnam/hn5003/2018		MW936128	Center for Research on Influenza Pathogenesis (CRIP), CEIRS Data Pr	unknown
a/duck/vietnam/hn4793/2018		MW936245	Center for Research on Influenza Pathogenesis (CRIP), CEIRS Data Pr	unknown
a/duck/vietnam/hn4797/2018		MW936101	Center for Research on Influenza Pathogenesis (CRIP), CEIRS Data Pr	unknown
a/duck/vietnam/hn4798/2018		MW936070	Center for Research on Influenza Pathogenesis (CRIP), CEIRS Data Pr	unknown
a/duck/vietnam/hn4792/2018		MW936299	Center for Research on Influenza Pathogenesis (CRIP), CEIRS Data Pr	unknown
a/duck/vietnam/hn5001/2018		MW936014	Center for Research on Influenza Pathogenesis (CRIP), CEIRS Data Pr	unknown

a/duck/vietnam/hn5955/2019		MW935270	Center for Research on Influenza Pathogenesis (CRIP), CEIRS Data Pr	unknown
a/duck/vietnam/hn5953/2019		MW935054	Center for Research on Influenza Pathogenesis (CRIP), CEIRS Data Pr	unknown
a/duck/vietnam/hn5958/2019		MW935188	Center for Research on Influenza Pathogenesis (CRIP), CEIRS Data Pr	unknown
a/duck/vietnam/hn5952/2019		MW935362	Center for Research on Influenza Pathogenesis (CRIP), CEIRS Data Pr	unknown
a/duck/vietnam/hn5956/2019		MW935282	Center for Research on Influenza Pathogenesis (CRIP), CEIRS Data Pr	unknown
a/duck/vietnam/hn5474/2019		MW935323	Center for Research on Influenza Pathogenesis (CRIP), CEIRS Data Pr	unknown
a/muscovy_duck/vietnam/hn5482/2019		MW935650	Center for Research on Influenza Pathogenesis (CRIP), CEIRS Data Pr	unknown
a/muscovy_duck/vietnam/hn5485/2019		MW935595	Center for Research on Influenza Pathogenesis (CRIP), CEIRS Data Pr	unknown
a/duck/vietnam/hn6471/2020		MW873066	Center for Research on Influenza Pathogenesis (CRIP), CEIRS Data Pr	unknown
a/duck/vietnam/hn6479/2020		MW872910	Center for Research on Influenza Pathogenesis (CRIP), CEIRS Data Pr	unknown
a/duck/vietnam/qn6586/2020		MW873887	Center for Research on Influenza Pathogenesis (CRIP), CEIRS Data Pr	unknown
a/duck/vietnam/qn6589/2020		MW873324	Center for Research on Influenza Pathogenesis (CRIP), CEIRS Data Pr	unknown
a/duck/vietnam/hn6617/2020		MW872800	Center for Research on Influenza Pathogenesis (CRIP), CEIRS Data Pr	unknown
a/muscovy_duck/vietnam/qn6297/2020		MW873924	Center for Research on Influenza Pathogenesis (CRIP), CEIRS Data Pr	unknown
a/muscovy_duck/vietnam/qn6305/2020		MW873329	Center for Research on Influenza Pathogenesis (CRIP), CEIRS Data Pr	unknown
a/duck/vietnam/hn5616/2019		MW934878	Center for Research on Influenza Pathogenesis (CRIP), CEIRS Data Pr	unknown
a/duck/vietnam/hn5620/2019		MW934734	Center for Research on Influenza Pathogenesis (CRIP), CEIRS Data Pr	unknown
a/muscovy_duck/vietnam/hn5481/2019		MW935421	Center for Research on Influenza Pathogenesis (CRIP), CEIRS Data Pr	unknown
a/muscovy_duck/vietnam/hn5489/2019		MW934869	Center for Research on Influenza Pathogenesis (CRIP), CEIRS Data Pr	unknown
a/muscovy_duck/viet_nam/qn-2479/2015		MK943454	J. Craig Venter Institute	unknown
a/duck/shimane/321101/2015	EPI855503		National Institute of Animal Health	National Institute of Animal Health
a/duck/vietnam/lbm814/2015		LC496112	Avian Zoonosis Research Center, Faculty of Agriculture; 4-101, Koyam	unknown
a/bean_goose/hubei/chenhu__xvi35-1/2016_h3n2	EPI737451		Wuhan Institute of Virology	State Key Laboratory of Virology and Wuhan Institut
a/duck/aichi/231019/2016	EPI866882		National Institute of Animal Health	unknown
a/duck/fukui/181019/2016	EPI866818		National Institute of Animal Health	unknown
a/duck/hunan/04.14_yygk431-p/2015_mixed_	EPI665893		Institute of Microbiology, Chinese Academy of Sciences	unknown
a/duck/shimane/321017/2015	EPI855865		National Institute of Animal Health	National Institute of Animal Health
a/duck/tottori/311018/2015	EPI855727		National Institute of Animal Health	National Institute of Animal Health
a/duck/kagoshima/ku-d46/2016	EPI1064637		Kagoshima University	unknown
a/wild_bird/south_korea/ku-05/2016	EPI2868526		Korea University	Korea University
a/wild_bird/south_korea/snu5/2016		OR999393	Korea University, Department of Pharmacy	unknown
a/duck/vietnam/hn5421/2019		MW934953	Center for Research on Influenza Pathogenesis (CRIP), CEIRS Data Pr	unknown
a/duck/vietnam/hn5426/2019		MW934945	Center for Research on Influenza Pathogenesis (CRIP), CEIRS Data Pr	unknown
a/duck/vietnam/hn6425/2020		MW873033	Center for Research on Influenza Pathogenesis (CRIP), CEIRS Data Pr	unknown
a/muscovy_duck/vietnam/hn5629/2019		MW935302	Center for Research on Influenza Pathogenesis (CRIP), CEIRS Data Pr	unknown
a/duck/vietnam/hn6212/2020		MW873163	Center for Research on Influenza Pathogenesis (CRIP), CEIRS Data Pr	unknown
a/muscovy_duck/vietnam/hn6220/2020		MW872988	Center for Research on Influenza Pathogenesis (CRIP), CEIRS Data Pr	unknown
a/duck/vietnam/hn6211/2020		MW873134	Center for Research on Influenza Pathogenesis (CRIP), CEIRS Data Pr	unknown
a/duck/vietnam/hn6219/2020		MW873153	Center for Research on Influenza Pathogenesis (CRIP), CEIRS Data Pr	unknown
a/muscovy_duck/viet_nam/qn-2615/2016		MK943494	J. Craig Venter Institute	unknown
a/duck/vietnam/lbm839/2015		LC496128	Avian Zoonosis Research Center, Faculty of Agriculture; 4-101, Koyam	unknown
a/anseriformes/anhui/tt41/2014		MN148472	Guangdong Ocean University, Agriculture college	unknown
a/anseriformes/anhui/xz4/2014		MN148488	Guangdong Ocean University, Agriculture college	unknown
a/anseriformes/anhui/xz9/2014		MN148496	Guangdong Ocean University, Agriculture college	unknown
a/anseriformes/anhui/xz3/2014		MN148480	Guangdong Ocean University, Agriculture college	unknown
a/anseriformes/anhui/xz1/2014		MN148464	Guangdong Ocean University, Agriculture college	unknown
a/duck/vietnam/lbm907/2016	EPI1583193		unknown	unknown
a/muscovy_duck/viet_nam/qn-2529/2015		MK943462	J. Craig Venter Institute	unknown
a/muscovy_duck/vietnam/lbm946/2017	EPI1583200		unknown	unknown
a/duck/vietnam/hn4931/2018		MW935597	Center for Research on Influenza Pathogenesis (CRIP), CEIRS Data Pr	unknown
a/duck/vietnam/hn4911/2018		MW936086	Center for Research on Influenza Pathogenesis (CRIP), CEIRS Data Pr	unknown

a/muscovy_duck/vietnam/hn4903/2018		MW936181	Center for Research on Influenza Pathogenesis (CRIP), CEIRS Data Pr	unknown
a/duck/vietnam/hn4917/2018		MW935914	Center for Research on Influenza Pathogenesis (CRIP), CEIRS Data Pr	unknown
a/muscovy_duck/vietnam/hn4910/2018		MW936165	Center for Research on Influenza Pathogenesis (CRIP), CEIRS Data Pr	unknown
a/duck/vietnam/ncvd-nd4v3p/2016	EPI1335783		Centers for Disease Control and Prevention	National Centre of Veterinary Diagnostics
a/wild_duck/shandong/w11221/2019	EPI2245758		Liaocheng University	College of Agronomy, Liaocheng University
a/duck/aichi/231013/2016	EPI866874		National Institute of Animal Health	unknown
a/duck/vietnam/hn6561/2020	EPI1974522		unknown	unknown
a/duck/vietnam/hn6569/2020	EPI1974460		unknown	unknown
a/duck/gunma/3/2016	EPI867672		National Institute of Animal Health	unknown
a/duck/gunma/4/2016	EPI867656		National Institute of Animal Health	unknown
a/rufous_turtle_dove/south_korea/knu18-19/2018	EPI3268155		unknown	Wonkwang University School of Medicine, Infection
a/duck/jiangsu/js1018/2019		OM527197	Yangzhou University, College of Veterinary Medicine	unknown
a/wild_duck/south_korea/knu2020-134/2020		OK253021	Wonkwang University, Zoonosis research center	unknown
a/green-winged_teal_anas_crecca_/south_korea/knu2019-57/2019		MW527117	Zoonosis Research Center, School of Medicine, Department of Infecti	unknown
a/duck/guangdong/1223/2019	EPI2594530		unknown	unknown
a/duck/shantou/1223/2019		OQ293429	The University of Hong Kong, School of Public Health	unknown
a/mallard_anas_platyrhynchos_/south_korea/knu2019-53/2019		MW527109	Zoonosis Research Center, School of Medicine, Department of Infecti	unknown
a/mallard_anas_platyrhynchos_/south_korea/nu2019-63/2019		MW391866	Zoonosis Research Center, School of Medicine, Department of Infecti	unknown
a/mallard_anas_platyrhynchos_/south_korea/knu2019-52/2019	EPI1902790		unknown	unknown
a/mallard_anas_platyrhynchos_/south_korea/knu2019-50/2019		MW486974	Zoonosis Research Center, School of Medicine, Department of Infecti	unknown
a/mallard_anas_platyrhynchos_/south_korea/knu2019-56/2019	EPI1902798		unknown	unknown
a/spot-billed_duck/south_korea/knu2020-105/2020		OK235631	Wonkwang University, Zoonosis research center	unknown
a/wild_duck/south_korea/knu2020-131/2020		PP746174	Wonkwang University School of Medicine, Infection Biology	unknown
a/wild_bird/south_korea/knu2020-77/2020	EPI1931666		unknown	unknown
a/wild_duck/south_korea/knu2020-74/2020		OK235640	Zoonosis Research Center, School of Medicine, Department of Infecti	unknown
a/spot-billed_duck/south_korea/knu2022-20/2022		PP746188	Wonkwang University School of Medicine, Infection Biology	unknown
a/duck/bangladesh/17d1961/2022	EPI3115919		Centers for Disease Control and Prevention	icddr,b International Centre for Diarrhoeal Disease
a/duck/bangladesh/17d1968/2022	EPI3115926		Centers for Disease Control and Prevention	icddr,b International Centre for Diarrhoeal Disease
a/equine/jilin/1/1989	EPI130279		unknown	unknown
a/psittacine/italy/2873/2000		GQ247846	Research Centre for Emerging Viral Infections, Medical Biotechnology	unknown
a/duck/siberia/100/2001		AB450457	Graduate School of Veterinary Medicine; kita-ku,kita18 nishi9, Sappo	unknown
a/chicken/nanchang/7-010/2000	EPI90336		unknown	unknown
a/quail/nanchang/7-026/2000		CY006014	St. Jude Children's Research Hospital, Hartwell Center for Bioinforma	unknown
a/aquatic_bird/korea/cn-1/2004		EU301210	Chungbuk National University, College of Medicine and Medical Rese	unknown
a/duck/vietnam/oie-2403/2009	EPI243476		unknown	unknown
a/chicken/wuxi/4859/2015	EPI1055297		Beijing Institute of Microbiology and Epidemiology	Beijing Institute of Microbiology and Epidemiology
a/duck/jiangsu/26/2004	EPI414138		unknown	unknown
a/duck/mongolia/181/2010	EPI1153583		unknown	unknown
a/ruddy_shelduck/mongolia/963v/2009		KF501068	School of Public Health, The University of Hong Kong	unknown
a/northern_shoveler/mongolia/957/2009		KF501095	School of Public Health, The University of Hong Kong	unknown
a/northern_shoveler/_mongolia/973/2009		KF501054	School of Public Health, The University of Hong Kong	unknown
a/ruddy_shelduck/mongolia/961v/2009		KF501086	School of Public Health, The University of Hong Kong	unknown
a/northern_shoveler/mongolia/977v/2009		KF501059	School of Public Health, The University of Hong Kong	unknown
a/goose/guangxi/020g/2009	EPI531636		unknown	unknown
a/pigeon/guangxi/020p/2009	EPI550305		unknown	unknown
a/duck/hunan/199/2014		KX121246	Lanzhou Veterinary Research Institute	unknown
a/duck/guangdong/04.23_dgqtsj126-o/2015_mixed_	EPI666605		Institute of Microbiology, Chinese Academy of Sciences	unknown
a/duck/guangdong/04.23_dgqtsj123-p/2015_mixed_	EPI666669		Institute of Microbiology, Chinese Academy of Sciences	unknown
a/duck/guangdong/04.23_dgqtsj122-p/2015_mixed_	EPI666309		Institute of Microbiology, Chinese Academy of Sciences	unknown
a/duck/guangdong/04.23_dgqtsj126-p/2015_mixed_	EPI666461		Institute of Microbiology, Chinese Academy of Sciences	unknown
a/duck/jiangsu/j3602/2014		KP767633	China Animal Health and Epidemiology Center, Laboratory of Avian Di	unknown

a/duck/guangdong/04.16_szlgwl009/2015_mixed_	EPI667261		Institute of Microbiology, Chinese Academy of Sciences	unknown
a/duck/china/402d22/2019		MW368584	Guangxi Veterinary Rerearch Institute, Guangxi Key Laboratory of Vete	unknown
a/duck/china/402d27/2019		MW368592	Guangxi Veterinary Rerearch Institute, Guangxi Key Laboratory of Vete	unknown
a/duck/jiangxi/01.08_ygcb023-p/2015_mixed_	EPI666221		Institute of Microbiology, Chinese Academy of Sciences	unknown
a/chicken/jiangxi/01.08_ygcb018-p/2015_h3n2_	EPI660871		Institute of Microbiology, Chinese Academy of Sciences	unknown
a/chicken/jiangxi/02.05_ygyxg001-p/2015_h3n2_	EPI660831		Institute of Microbiology, Chinese Academy of Sciences	unknown
a/duck/jiangxi/01.14_ncjd027-p/2015_mixed_	EPI666149		Institute of Microbiology, Chinese Academy of Sciences	unknown
a/duck/jiangxi/01.14_ncjd031-p/2015_mixed_	EPI666197		Institute of Microbiology, Chinese Academy of Sciences	unknown
a/duck/jiangxi/01.14_ncjd058-p/2015_mixed_	EPI666229		Institute of Microbiology, Chinese Academy of Sciences	unknown
a/duck/jiangxi/01.14_ncjd059-p/2015_mixed_	EPI666253		Institute of Microbiology, Chinese Academy of Sciences	unknown
a/duck/jiangxi/01.14_ncjd028-p/2015_h3n8_	EPI660983		Institute of Microbiology, Chinese Academy of Sciences	unknown
a/duck/jiangxi/01.14_ncjd057-p/2015_mixed_	EPI666261		Institute of Microbiology, Chinese Academy of Sciences	unknown
a/duck/jiangxi/01.14_ncjd035-p/2015_h3n2_	EPI660895		Institute of Microbiology, Chinese Academy of Sciences	unknown
a/duck/jiangxi/01.14_ncjd038-p/2015_h3n2_	EPI660887		Institute of Microbiology, Chinese Academy of Sciences	unknown
a/duck/jiangxi/01.14_ncjd033-p/2015_mixed_	EPI666165		Institute of Microbiology, Chinese Academy of Sciences	unknown
a/aquatic_bird/jeonju/kn4/2005		MW547664	Chungbuk National University, College of Medicine and Medical Rese	unknown
a/baikal_teal/shanghai/sh-89/2013		KJ907503	Academy of Military Medical Sciences, Department of Virology Resea	unknown
a/baikal_teal/shanghai/sh-101/2013		KJ907511	Academy of Military Medical Sciences, Department of Virology Resea	unknown
a/baikal_teal/shanghai/sh-104/2013		KJ907727	Academy of Military Medical Sciences, Department of Virology Resea	unknown
a/muscovy_duck/vietnam/lbm721/2014	EPI596568		unknown	unknown
a/muscovy_duck/vietnam/lbm728/2014	EPI590059		unknown	unknown
a/duck/viet_nam/hn-2632/2016	EPI1576660		unknown	unknown
a/muscovy_duck/viet_nam/hn-2227/2015		MK943074	J. Craig Venter Institute	unknown
a/muscovy_duck/viet_nam/hn-2229/2015		MK943051	J. Craig Venter Institute	unknown
a/chicken/cambodia/prorsam/010401/2011/h3n8	EPI702905		Swedish Veterinary Agency (SVA)	National Veterinary Research Institute
a/pigeon/anhui/08/2013		KJ579956	Huazhong Agricultural University, State Key Laboratory of Agricultural	unknown
a/duck/shanghai/sh3/2013		KM222572	Shanghai Animal Disease Control Center	unknown
a/duck/jiangsu/j1438/2014		KP765951	China Animal Health and Epidemiology Center, Laboratory of Avian Di	unknown
a/chicken/shanghai/lpm2/2013		KM222548	Shanghai Animal Disease Control Center	unknown
a/duck/shanghai/02/2014		KR703241	Shanghai Animal Disease Control Center, Veterinary Disease Diagnos	unknown
a/chicken/jiangsu/12.30_wznhq013-p/2014_mixed_	EPI666949		Institute of Microbiology, Chinese Academy of Sciences	unknown
a/chicken/nanjing/b854-2/2011		KU158890	Chinese Academy of Agricultural Sciences, Shanghai Veterinary Resea	unknown
a/duck/nanjing/a1591-1/2010	EPI774952		unknown	unknown
a/mallard/xuyi/14/2015	EPI895479		unknown	unknown
a/duck/anhui/a1456/2014		KP765884	China Animal Health and Epidemiology Center, Laboratory of Avian Di	unknown
a/duck/anhui/a1785/2014		KP765903	China Animal Health and Epidemiology Center, Laboratory of Avian Di	unknown
a/duck/anhui/a1835/2014		KP765928	China Animal Health and Epidemiology Center, Laboratory of Avian Di	unknown
a/duck/anhui/a1480/2014		KP765896	China Animal Health and Epidemiology Center, Laboratory of Avian Di	unknown
a/duck/anhui/a1474/2014		KP765893	China Animal Health and Epidemiology Center, Laboratory of Avian Di	unknown
a/duck/anhui/a1792/2014		KP765909	China Animal Health and Epidemiology Center, Laboratory of Avian Di	unknown
a/duck/anhui/a1787/2014		KP765905	China Animal Health and Epidemiology Center, Laboratory of Avian Di	unknown
a/duck/anhui/a1804/2014		KP765913	China Animal Health and Epidemiology Center, Laboratory of Avian Di	unknown
a/duck/anhui/a1810/2014		KP765916	China Animal Health and Epidemiology Center, Laboratory of Avian Di	unknown
a/duck/anhui/a1826/2014		KP765922	China Animal Health and Epidemiology Center, Laboratory of Avian Di	unknown
a/duck/anhui/a1467/2014		KP765891	China Animal Health and Epidemiology Center, Laboratory of Avian Di	unknown
a/duck/anhui/a1789/2014		KP765906	China Animal Health and Epidemiology Center, Laboratory of Avian Di	unknown
a/duck/anhui/a1843/2014		KP765933	China Animal Health and Epidemiology Center, Laboratory of Avian Di	unknown
a/duck/anhui/a1870/2014		KP765939	China Animal Health and Epidemiology Center, Laboratory of Avian Di	unknown
a/duck/anhui/a1830/2014		KP765924	China Animal Health and Epidemiology Center, Laboratory of Avian Di	unknown
a/duck/anhui/a1840/2014	EPI708180		unknown	unknown
a/duck/anhui/a1878/2014	EPI708199		unknown	unknown

a/duck/anhui/a1862/2014		KP765937	China Animal Health and Epidemiology Center, Laboratory of Avian Di	unknown
a/duck/anhui/a1865/2014	EPI708187		unknown	unknown
a/duck/anhui/a1888/2014		KP765947	China Animal Health and Epidemiology Center, Laboratory of Avian Di	unknown
a/duck/anhui/a1877/2014	EPI708195		unknown	unknown
a/duck/anhui/a1819/2014		KP765920	China Animal Health and Epidemiology Center, Laboratory of Avian Di	unknown
a/duck/anhui/a1790/2014		KP765907	China Animal Health and Epidemiology Center, Laboratory of Avian Di	unknown
a/duck/anhui/a1472/2014		KP765892	China Animal Health and Epidemiology Center, Laboratory of Avian Di	unknown
a/duck/anhui/a1808/2014		KP765914	China Animal Health and Epidemiology Center, Laboratory of Avian Di	unknown
a/duck/anhui/a1809/2014		KP765915	China Animal Health and Epidemiology Center, Laboratory of Avian Di	unknown
a/aquatic_bird/korea/cn-4/2006		EU301213	Chungbuk National University, College of Medicine and Medical Rese	unknown
a/duck/vietnam/g119/2006		AB593431	Faculty of Life Sciences; Motoyama, Kamigamo, Kita-Ku, Kyoto, Kyoto	unknown
a/aquatic_bird/jeonju/338/2008		MW547663	Chungbuk National University, College of Medicine and Medical Rese	unknown
a/whooper_swan/mongolia/1-14/2007		JN029550	National Veterinary Research and Quarantine Service, Avian Disease	unknown
a/swan/shimane/227/01	EPI3017		unknown	unknown
a/common_teal/mongolia/1906/2011		KF501058	School of Public Health, The University of Hong Kong	unknown
a/duck/jiangsu/4/2010	EPI414146		unknown	unknown
a/duck/bangladesh/1798/2010	EPI540295		Centers for Disease Control and Prevention	icddr,b International Centre for Diarrhoeal Disease
a/duck/bangladesh/1800/2010	EPI540303		Centers for Disease Control and Prevention	icddr,b International Centre for Diarrhoeal Disease
a/duck/beijing/44/04	EPI234655		unknown	unknown
a/duck/beijing/61/05	EPI232402		unknown	unknown
a/duck/beijing/40/04	EPI234648		unknown	unknown
a/duck/beijing/33/04	EPI234641		unknown	unknown
a/duck/beijing/59/05	EPI232416		unknown	unknown
a/duck/beijing/56/05	EPI232409		unknown	unknown
a/duck/bangladesh/1025/2011	EPI540287		Centers for Disease Control and Prevention	icddr,b International Centre for Diarrhoeal Disease
a/duck/bangladesh/1822/2011	EPI540279		Centers for Disease Control and Prevention	icddr,b International Centre for Diarrhoeal Disease
a/garganey/altai/1216/2007		CY049780	State Research Center of Virology and Biotechnology "Vector"	unknown
a/mallard/altai/1208/2007		CY049764	State Research Center of Virology and Biotechnology "Vector"	unknown
a/gadwall/altai/1325/2007	EPI222063		unknown	unknown
a/mallard/mongolia/2377/2011		KF501080	School of Public Health, The University of Hong Kong	unknown
a/eurasian_curlew/zhuanghe/zh-65/2013		KJ907535	Academy of Military Medical Sciences, Department of Virology Resear	unknown
a/eurasian_curlew/zhuanghe/zh-47/2013		KJ907519	Academy of Military Medical Sciences, Department of Virology Resear	unknown
a/eurasian_curlew/zhuanghe/zh-64/2013		KJ907527	Academy of Military Medical Sciences, Department of Virology Resear	unknown
a/duck/mongolia/200/2015	EPI704356		unknown	unknown
a/bar-headed_goose__anser_indicus_/india/dbc1-12a/2016		MW036340	National Institute of High Security Animal Diseases, Pathogenomics la	unknown
a/gadwall/buryatia/2226/2019	EPI1638508		National Institute of Animal Health	Research Institute of Experimental and Clinical Me
a/duck/mongolia/2021-mg05/2021		OP658923	Zoonosis Research Center, School of Medicine, Wonkwang University	unknown
a/duck/mongolia/2021-mg09/2021		OP704037	Zoonosis Research Center, School of Medicine, Department of Infecti	unknown
a/common_shelduck/mongolia/2106/2011		KF501072	School of Public Health, The University of Hong Kong	unknown
a/wild_bird/mongolia/1782/2011		KF501078	School of Public Health, The University of Hong Kong	unknown
a/duck/mongolia/675/2015		LC132936	Graduate School of Veterinary Medicine; Kita-18 Nishi-9, Kita-ku, Sap	unknown
a/duck/mongolia/20/2015		LC108112	Graduate School of Veterinary Medicine; Kita-18 Nishi-9, Kita-ku, Sap	unknown
a/duck/mongolia/637/2019		MT020214	Hokkaido University, Faculty of Veterinary Medicine	unknown
a/duck/mongolia/619/2019	EPI1777525		unknown	unknown
a/wild_duck/shandong/w1923/2017	EPI2245792		Liaocheng University	College of Agronomy, Liaocheng University
a/wild_duck/shandong/w2003/2017	EPI2245800		Liaocheng University	College of Agronomy, Liaocheng University
a/wild_duck/shandong/w1895/2017	EPI2245784		Liaocheng University	College of Agronomy, Liaocheng University
a/duck/mongolia/144/2005		LC339873	Faculty of Veterinary Medicine; kita18 nishi9, kita-ku, Sapporo, Hokka	unknown
a/duck/shanghai/120-1/2009		KU158888	Chinese Academy of Agricultural Sciences, Shanghai Veterinary Resea	unknown
a/mallard/mongolia/1551/2010		KF501074	School of Public Health, The University of Hong Kong	unknown
a/mallard_/mongolia/1551/2010	EPI548449		unknown	unknown

a/red_crested_pochard/mongolia/1915/2006	EPI230013		unknown	unknown
a/ruddy_shelduck/mongolia/2-29/2007	EPI387486		unknown	unknown
a/domestic_green-winged_teal/hunan/2036/2007	EPI439827		unknown	unknown
a/whooper_swan/mongolia/1-25/2007	EPI387407		unknown	unknown
a/ruddy_shelduck/mongolia/1-26/2007		JN029588	National Veterinary Research and Quarantine Service, Avian Disease	unknown
a/duck/mongolia/621/2010		LC367450	Faculty of Veterinary Medicine; kita18 nishi9, kita-ku, Sapporo, Hokka	unknown
a/duck/thailand/cu-7518c/2010		JX307139	Veterinary Public Health, Chulalongkorn University	unknown
a/duck/thailand/cu-7519c/2010		JX307235	Veterinary Public Health, Chulalongkorn University	unknown
a/ruddy_shelduck/mongolia/881v/2009		KF501084	School of Public Health, The University of Hong Kong	unknown
a/northern_shoveler/mongolia/992v/2009		KF501063	School of Public Health, The University of Hong Kong	unknown
a/ruddy_shelduck/mongolia/882v/2009		KF501089	School of Public Health, The University of Hong Kong	unknown
a/ruddy_shelduck/mongolia/1787/2011		KF501073	School of Public Health, The University of Hong Kong	unknown
a/duck/vietnam/lbm235/2012		AB812573	Avian Zoonosis Research Center,Faculty of Agriculture; 4-101 Koyama	unknown
a/muscovy_duck/vietnam/lbm240/2012	EPI432692		unknown	unknown
a/common_shelduck/mongolia/2185/2011		KF501057	School of Public Health, The University of Hong Kong	unknown
a/duck/mongolia/139/2010		LC339865	Faculty of Veterinary Medicine; kita18 nishi9, kita-ku, Sapporo, Hokka	unknown
a/velvet_scoter/mongolia/969v/2009		KF501088	School of Public Health, The University of Hong Kong	unknown
a/duck/mongolia/131/2010		LC339857	Faculty of Veterinary Medicine; kita18 nishi9, kita-ku, Sapporo, Hokka	unknown
a/duck/mongolia/62/2013		LC367442	Faculty of Veterinary Medicine; kita18 nishi9, kita-ku, Sapporo, Hokka	unknown
a/duck/mongolia/122/2015		LC132904	Graduate School of Veterinary Medicine; Kita-18 Nishi-9, Kita-ku, Sap	unknown
a/duck/mongolia/129/2015		LC132920	Graduate School of Veterinary Medicine; Kita-18 Nishi-9, Kita-ku, Sap	unknown
a/duck/bangladesh/1772/2010	EPI540271		Centers for Disease Control and Prevention	icddr,b International Centre for Diarrhoeal Disease
a/duck/thailand/cu-11671c/2011		KT318506	Center of Emerging and Infectious Diseases in Animals (CU-EIDAs), Fa	unknown
a/duck/thailand/cu-11676c/2011		KT318514	Center of Emerging and Infectious Diseases in Animals (CU-EIDAs), Fa	unknown
a/duck/thailand/cu-11682c/2011		KT318521	Center of Emerging and Infectious Diseases in Animals (CU-EIDAs), Fa	unknown
a/duck/thailand/cu-11679t/2011		KT318519	Center of Emerging and Infectious Diseases in Animals (CU-EIDAs), Fa	unknown
a/duck/thailand/cu-11686t/2011		KT318523	Center of Emerging and Infectious Diseases in Animals (CU-EIDAs), Fa	unknown
a/duck/bangladesh/1574/2009	EPI540311		Centers for Disease Control and Prevention	icddr,b International Centre for Diarrhoeal Disease
a/duck/bangladesh/1575/2009	EPI540327		Centers for Disease Control and Prevention	icddr,b International Centre for Diarrhoeal Disease
a/duck/bangladesh/1576/2009	EPI540319		Centers for Disease Control and Prevention	icddr,b International Centre for Diarrhoeal Disease
a/pintail/chany/198/2016	EPI925980		WHO National Influenza Centre Russian Federation	Research Institute of Experimental and Clinical Me
a/mallard/chany/185/2016	EPI925972		WHO National Influenza Centre Russian Federation	Research Institute of Experimental and Clinical Me
a/teal/chany/135/2016	EPI925964		WHO National Influenza Centre Russian Federation	Research Institute of Experimental and Clinical Me
a/duck/bangladesh/31019/2016	EPI1099154		unknown	unknown
a/duck/bangladesh/30828/2016	EPI1098796		unknown	unknown
a/duck/bangladesh/30824/2016	EPI1099048		unknown	unknown
a/duck/bangladesh/30827/2016	EPI1099140		unknown	unknown
a/duck/mongolia/146/2010	EPI1153558		unknown	unknown
a/duck/mongolia/53/2011		LC367410	Faculty of Veterinary Medicine; kita18 nishi9, kita-ku, Sapporo, Hokka	unknown
a/duck/mongolia/646/2010		LC367466	Faculty of Veterinary Medicine; kita18 nishi9, kita-ku, Sapporo, Hokka	unknown
a/duck/mongolia/593/2010		LC367434	Faculty of Veterinary Medicine; kita18 nishi9, kita-ku, Sapporo, Hokka	unknown
a/duck/mongolia/672/2010		LC367490	Faculty of Veterinary Medicine; kita18 nishi9, kita-ku, Sapporo, Hokka	unknown
a/duck/mongolia/30/2011		LC349383	Faculty of Veterinary Medicine; kita18 nishi9, kita-ku, Sapporo, Hokka	unknown
a/duck/mongolia/120/2011		LC339803	Faculty of Veterinary Medicine; kita18 nishi9, kita-ku, Sapporo, Hokka	unknown
a/duck/mongolia/487/2011		LC367378	Faculty of Veterinary Medicine; kita18 nishi9, kita-ku, Sapporo, Hokka	unknown
a/duck/mongolia/103/2011		LC339755	Faculty of Veterinary Medicine; kita18 nishi9, kita-ku, Sapporo, Hokka	unknown
a/duck/mongolia/1047/2009	EPI1153538		unknown	unknown
a/duck/kerala/dutr1411100009/2014	EPI1889826		unknown	unknown
a/chicken/kerala/chcl1411100011/2014		MT272373	ICAR-National Institute of High Security Animal Disease, Diagnostic I	unknown
a/duck/kerala/ducl1411100010/2014	EPI1889802		unknown	unknown
a/duck/mongolia/395/2011		LC349423	Faculty of Veterinary Medicine; kita18 nishi9, kita-ku, Sapporo, Hokka	unknown

a/duck/mongolia/402/2011		LC349439	Faculty of Veterinary Medicine; kita18 nishi9, kita-ku, Sapporo, Hokka	unknown
a/duck/mongolia/398/2011		LC349431	Faculty of Veterinary Medicine; kita18 nishi9, kita-ku, Sapporo, Hokka	unknown
a/duck/mongolia/394/2011	EPI1134072		unknown	unknown
a/duck/mongolia/405/2011	EPI1175810		unknown	unknown
a/mallard/mongolia/1581/2010		KF501067	School of Public Health, The University of Hong Kong	unknown
a/duck/mongolia/248/2010	EPI1134019		unknown	unknown
a/duck/mongolia/oie-7457/2011		AB701296	Graduate School of Veterinary Medicine; kita-ku,kita18 nishi9, Sappor	unknown
a/duck/mongolia/235/2014		LC349311	Faculty of Veterinary Medicine; kita18 nishi9, kita-ku, Sapporo, Hokka	unknown
a/duck/mongolia/27/2011	EPI1133997		unknown	unknown
a/duck/mongolia/405/2015	EPI704380		unknown	unknown
a/duck/mongolia/572/2015		LC132928	Graduate School of Veterinary Medicine; Kita-18 Nishi-9, Kita-ku, Sap	unknown
a/duck/mongolia/mn18-1/2018		MW487361	Zoonosis Research Center, School of Medicine, Department of Infecti	unknown
a/gadwall/buryatia/2209/2019	EPI1638492		National Institute of Animal Health	Research Institute of Experimental and Clinical Me
a/duck/mongolia/667/2019		MT020230	Hokkaido University, Faculty of Veterinary Medicine	unknown
a/duck/mongolia/398/2018	EPI1818084		unknown	unknown
a/mallard/buryatia/114/2018	EPI1353344		National Institute of Animal Health	Research Institute of Experimental and Clinical Me
a/duck/mongolia/210/2018	EPI1818044		unknown	unknown
a/duck/mongolia/314/2018		MW188589	Hokkaido University, Faculty of Veterinary Medicine	unknown
a/duck/mongolia/116/2015		LC121252	Graduate School of Veterinary Medicine; Kita-18 Nishi-9, Kita-ku, Sap	unknown
a/duck/mongolia/124/2015		LC132912	Graduate School of Veterinary Medicine; Kita-18 Nishi-9, Kita-ku, Sap	unknown
a/duck/mongolia/2021-mg03/2021	EPI2257138		unknown	unknown
a/duck/mongolia/2021-mg04/2021	EPI2257114		unknown	unknown
a/duck/mongolia/mn18-2/2018	EPI1903599		unknown	unknown
a/duck/mongolia/2021-mg11/2021	EPI2257504		unknown	unknown
a/duck/mongolia/2021-mg12/2021		OP704105	Zoonosis Research Center, School of Medicine, Department of Infecti	unknown
a/duck/mongolia/mn18-5/2018	EPI1903607		unknown	unknown
a/duck/mongolia/mn18-10/2018	EPI1903615		unknown	unknown
a/duck/mongolia/2021-mg07/2021		OP658940	Zoonosis Research Center, School of Medicine, Wonkwang University	unknown
a/pet_bird/hong_kong/1559/99		AJ427304	Chin P., Department of Microbiology, The University of Hong Kong, Uni	unknown
a/aquatic_bird/korea/kn-5/2006	EPI126975		unknown	unknown
a/whooper_swan/mongolia/1-21/2007	EPI387394		unknown	unknown
a/duck/hokkaido/17/2015		LC332531	Faculty of Veterinary Medicine; kita18 nishi9, kita-ku, Sapporo, Hokka	unknown
a/duck/hokkaido/13/2015		LC332507	Faculty of Veterinary Medicine; kita18 nishi9, kita-ku, Sapporo, Hokka	unknown
a/duck/hokkaido/20/2015		LC339523	Faculty of Veterinary Medicine; kita18 nishi9, kita-ku, Sapporo, Hokka	unknown
a/duck/hokkaido/14/2015		LC332515	Faculty of Veterinary Medicine; kita18 nishi9, kita-ku, Sapporo, Hokka	unknown
a/duck/hokkaido/15/2015	EPI1098328		unknown	unknown
a/duck/hokkaido/12/2015		LC332499	Faculty of Veterinary Medicine; kita18 nishi9, kita-ku, Sapporo, Hokka	unknown
a/duck/hokkaido/w70/2005		LC490208	Faculty of Veterinary Medicine; Kita18 Nishi9, Kita-ku, Sapporo, Hokk	unknown
a/duck/korea/u5-2/2007	EPI326852		unknown	unknown
a/mallard/south_korea/n07-0347/2007	EPI1790694		unknown	unknown
a/american_green-winged_teal/alaska/44419-342/2008		JX080732	U.S. Geological Survey, Alaska Science Center	unknown
a/northern_pintail/alaska/44500-075/2009		JX080770	U.S. Geological Survey, Alaska Science Center	unknown
a/american_green-winged_teal/alaska/44493-579/2009		JX080734	U.S. Geological Survey, Alaska Science Center	unknown
a/northern_pintail/alaska/44500-089/2009		JX080772	U.S. Geological Survey, Alaska Science Center	unknown
a/american_green-winged_teal/alaska/44419-233/2008	EPI442037		unknown	unknown
a/american_green-winged_teal/alaska/44419-209/2008	EPI442036		unknown	unknown
a/northern_pintail/alaska/44419-062/2008		GU143860	USGS - Alaska Science Center	unknown
a/northern_pintail/alaska/44419-104/2008		GU143859	USGS - Alaska Science Center	unknown
a/green-winged_teal/alaska/794/2012		KY130931	U.S. Geological Survey, Alaska Science Center	unknown
a/american_green-winged_teal/interior_alaska/10bm08222r0/2010		CY143556	on behalf of JCVI/Massachusetts Institute of Technology/NCBI, Natior	unknown
a/mallard/alberta/612/2015		MF613669	St. Jude Center of Excellence for Influenza Research and Surveillance	unknown

a/mallard/alberta/67/2015		MF046425	St. Jude Center of Excellence for Influenza Research and Surveillance	unknown
a/mallard/alberta/365/2015		MF046187	St. Jude Center of Excellence for Influenza Research and Surveillance	unknown
a/mallard/alberta/15/2015		MF046421	St. Jude Center of Excellence for Influenza Research and Surveillance	unknown
a/mallard/alberta/131/2015		MF613922	St. Jude Center of Excellence for Influenza Research and Surveillance	unknown
a/ruddy_turnstone/delaware_bay/368/2016	EPI1071745		unknown	unknown
a/blue-winged_teal/alberta/164/2017	EPI1304059		unknown	unknown
a/green-winged_teal/alberta/426/2017	EPI1299465		unknown	unknown
a/mallard/alberta/425/2017	EPI1299479		unknown	unknown
a/green-winged_teal/alberta/427/2017	EPI1299883		unknown	unknown
a/mallard/alberta/411/2017	EPI1299396		unknown	unknown
a/green-winged_teal/alberta/415/2017	EPI1299481		unknown	unknown
a/green-winged_teal/alberta/430/2017	EPI1299710		unknown	unknown
a/mallard/alberta/328/2017		MH637395	St. Jude Center of Excellence for Influenza Research and Surveillance	unknown
a/green-winged_teal/alberta/432/2017	EPI1299869		unknown	unknown
a/mallard/alberta/586/2017		MH932521	St. Jude Center of Excellence for Influenza Research and Surveillance	unknown
a/mallard_duck/alberta/358/2019		MT624534	St. Jude Center of Excellence for Influenza Research and Surveillance	unknown
a/green-winged_teal/alberta/428/2017	EPI1299628		unknown	unknown
a/blue-winged_teal/alberta/162/2017	EPI1304332		unknown	unknown
a/chicken/guangdong/01/2013		KJ917390	College of Veterinary Medicine, South China Agricultural University, C	unknown
a/mallard/sanjiang/90/2006		CY100443	Northeast Forestry University	unknown
a/duck/tsukuba/41/2005		AB669134	Research Team for Zoonotic Diseases; 3-1-5 Kannondai, Tsukuba, Iba	unknown
a/duck/tsukuba/41/2005_h3n1__	EPI356627		unknown	unknown
a/aquatic_bird/gunsan/cn3/2005		MW547660	Chungbuk National University, College of Medicine and Medical Rese	unknown
a/aquatic_bird/korea/cn-3/2005	EPI126959		unknown	unknown
a/duck/shiga/10413-24/2006		AB537453	Research Team for Zoonotic Diseases; Kannondai 3-1-5, Tsukuba, Iba	unknown
a/duck/chiba/14/2006		AB537463	Research Team for Zoonotic Diseases; Kannondai 3-1-5, Tsukuba, Iba	unknown
a/duck/tsukuba/38/2006		AB537452	Research Team for Zoonotic Diseases; Kannondai 3-1-5, Tsukuba, Iba	unknown
a/duck/chiba/31/2006		AB537466	Research Team for Zoonotic Diseases; Kannondai 3-1-5, Tsukuba, Iba	unknown
a/duck/chiba/44/2006		AB537468	Research Team for Zoonotic Diseases; Kannondai 3-1-5, Tsukuba, Iba	unknown
a/aquatic_bird/gunsan/146/2006		MW547662	Chungbuk National University, College of Medicine and Medical Rese	unknown
a/chicken/laos/a0573/2007		CY040966	Department of Infectious Diseases, St. Jude Children's Research Hos	unknown
a/duck/shimane/321006/2014	EPI855647		National Institute of Animal Health	National Institute of Animal Health
a/duck/chiba/20/2006		AB537464	Research Team for Zoonotic Diseases; Kannondai 3-1-5, Tsukuba, Iba	unknown
a/duck/chiba/20/06	EPI1139878		National Institute of Animal Health	National Institute of Animal Health
a/avian/japan/8ki0162/2008	EPI299266		unknown	unknown
a/avian/japan/8ki0180/2008		CY080215	on behalf of JCVI/University of Alaska Fairbanks/NCBI, National Cente	unknown
a/avian/japan/8ki0102/2008		CY079259	on behalf of JCVI/University of Alaska Fairbanks/NCBI, National Cente	unknown
a/duck/saitama/3/2009		AB531447	National Agriculture and Food Research Organization , Research Tea	unknown
a/duck/saitama/4/2009		AB531448	National Agriculture and Food Research Organization , Research Tea	unknown
a/duck/saitama/2/2009		AB531446	National Agriculture and Food Research Organization , Research Tea	unknown
a/duck/vietnam/lbm553/2013		LC028152	Avian Zoonosis Research Center; 4-101 Koyama-cho Minami, Tottori,	unknown
a/muscovy_duck/quang_ninh/131/2013		AB981450	Avian Zoonosis Research Center, Faculty of Agriculture; 4-101 Koyam	unknown
a/baikal_teal/xianghai/xh-28c/2012		KJ907495	Academy of Military Medical Sciences, Department of Virology Resea	unknown
a/duck/vietnam/lbm559/2013	EPI596491		unknown	unknown
a/duck/quang_ninh/14/2013	EPI596634		unknown	unknown
a/duck/quang_ninh/90c112/2013	EPI590080		unknown	unknown
a/duck/tsukuba/18/2008		AB537469	Research Team for Zoonotic Diseases; Kannondai 3-1-5, Tsukuba, Iba	unknown
a/duck/tsukuba/19/2008		AB537470	Research Team for Zoonotic Diseases; Kannondai 3-1-5, Tsukuba, Iba	unknown
a/duck/hokkaido/w4/2010		LC339683	Faculty of Veterinary Medicine; kita18 nishi9, kita-ku, Sapporo, Hokka	unknown
a/avian/japan/8ki0040/2008	EPI299314		unknown	unknown
a/avian/japan/8ki0150/2008		CY079235	on behalf of JCVI/University of Alaska Fairbanks/NCBI, National Cente	unknown

a/avian/japan/8ki0068/2008	EPI299306		unknown	unknown
a/avian/japan/8ki0067/2008	EPI300182		unknown	unknown
a/avian/japan/8ki0129/2008		CY079251	on behalf of JCVI/University of Alaska Fairbanks/NCBI, National Cente	unknown
a/duck/tsukuba/65/2008		AB537471	Research Team for Zoonotic Diseases; Kannondai 3-1-5, Tsukuba, Ibar	unknown
a/duck/chiba/4/2008		AB537474	Research Team for Zoonotic Diseases; Kannondai 3-1-5, Tsukuba, Ibar	unknown
a/duck/chiba/3/2008		AB537473	Research Team for Zoonotic Diseases; Kannondai 3-1-5, Tsukuba, Ibar	unknown
a/duck/chiba/7/2008		AB537475	Research Team for Zoonotic Diseases; Kannondai 3-1-5, Tsukuba, Ibar	unknown
a/duck/chiba/33/2008	EPI1177652		National Institute of Animal Health	National Institute of Animal Health
a/duck/chiba/34/2008	EPI1177724		National Institute of Animal Health	National Institute of Animal Health
a/duck/chiba/13/2008		AB537476	Research Team for Zoonotic Diseases; Kannondai 3-1-5, Tsukuba, Ibar	unknown
a/duck/chiba/15/2008		AB537477	Research Team for Zoonotic Diseases; Kannondai 3-1-5, Tsukuba, Ibar	unknown
a/duck/chiba/1/2008		AB537472	Research Team for Zoonotic Diseases; Kannondai 3-1-5, Tsukuba, Ibar	unknown
a/northern_pintail/alaska/993/2011		KY130763	U.S. Geological Survey, Alaska Science Center	unknown
a/common_teal/russia_primorje/113t/2020	EPI1849845		National Institute of Animal Health	Research Institute of Experimental and Clinical Me
a/northern_pintail/russia_primorje/96t/2020	EPI1850500		National Institute of Animal Health	Research Institute of Experimental and Clinical Me
a/baikal_teal/shanghai/sh-90/2013		KJ907543	Academy of Military Medical Sciences, Department of Virology Resear	unknown
a/northern_pintail/alaska/496/2012		KY130827	U.S. Geological Survey, Alaska Science Center	unknown
a/duck/saga/411008/2014	EPI855543		National Institute of Animal Health	National Institute of Animal Health
a-duck-ibaraki-1-2016-e1_s1	EPI866753		National Institute of Animal Health	unknown
a/duck/ibaraki/1/2016	EPI1105861		National Institute of Animal Health	National Institute of Animal Health
a/duck/hokkaido/91/2014	EPI1151628		unknown	unknown
a/duck/hokkaido/102/2014	EPI1153524		unknown	unknown
a/duck/hokkaido/103/2014		LC339747	Faculty of Veterinary Medicine; kita18 nishi9, kita-ku, Sapporo, Hokka	unknown
a/duck/hokkaido/10/2015	EPI1153517		unknown	unknown
a/wild_duck/shandong/w6252/2019	EPI2245816		Liaocheng University	College of Agronomy, Liaocheng University
a/garganey/yakutia/94/2020	EPI1848267		National Institute of Animal Health	Research Institute of Experimental and Clinical Me
a/garganey/yakutia/98/2020	EPI1848275		National Institute of Animal Health	Research Institute of Experimental and Clinical Me
a/common_teal/yakutia/128/2020	EPI1848235		National Institute of Animal Health	Research Institute of Experimental and Clinical Me
a/european_wigeon/yakutia/55/2020	EPI1848179		National Institute of Animal Health	Research Institute of Experimental and Clinical Me
a/common_teal/yakutia/59x/2020	EPI1848251		National Institute of Animal Health	Research Institute of Experimental and Clinical Me
a/common_teal/yakutia/60x/2020	EPI1848259		National Institute of Animal Health	Research Institute of Experimental and Clinical Me
a/common_teal/yakutia/57x/2020	EPI1848243		National Institute of Animal Health	Research Institute of Experimental and Clinical Me
a/common_teal/yakutia/49/2020	EPI1848171		National Institute of Animal Health	Research Institute of Experimental and Clinical Me
a/northern_pintail/yakutia/14/2020	EPI1848211		National Institute of Animal Health	Research Institute of Experimental and Clinical Me
a/mallard/yakutia/21/2020	EPI1848195		National Institute of Animal Health	Research Institute of Experimental and Clinical Me
a/shoveler/yakutia/25/2020	EPI1848219		National Institute of Animal Health	Research Institute of Experimental and Clinical Me
a/mallard/yakutia/11/2020	EPI1848187		National Institute of Animal Health	Research Institute of Experimental and Clinical Me
a/shoveler/yakutia/65/2020	EPI1848227		National Institute of Animal Health	Research Institute of Experimental and Clinical Me
a/duck/chiba/10/2006		AB537462	Research Team for Zoonotic Diseases; Kannondai 3-1-5, Tsukuba, Ibar	unknown
a/duck/chiba/24/2006		AB537465	Research Team for Zoonotic Diseases; Kannondai 3-1-5, Tsukuba, Ibar	unknown
a/duck/chiba/24/06	EPI1139805		National Institute of Animal Health	National Institute of Animal Health
a/duck/chiba/4/2006		AB537456	Research Team for Zoonotic Diseases; Kannondai 3-1-5, Tsukuba, Ibar	unknown
a/duck/chiba/4/06	EPI1139846		National Institute of Animal Health	National Institute of Animal Health
a/duck/chiba/3/2006		AB537455	Research Team for Zoonotic Diseases; Kannondai 3-1-5, Tsukuba, Ibar	unknown
a/duck/chiba/3/06	EPI1139854		National Institute of Animal Health	National Institute of Animal Health
a/duck/chiba/7/2006		AB537459	Research Team for Zoonotic Diseases; Kannondai 3-1-5, Tsukuba, Ibar	unknown
a/duck/chiba/10/06	EPI1139814		National Institute of Animal Health	National Institute of Animal Health
a/duck/chiba/5/06	EPI1139838		National Institute of Animal Health	National Institute of Animal Health
a/duck/chiba/2/2006		AB537454	Research Team for Zoonotic Diseases; Kannondai 3-1-5, Tsukuba, Ibar	unknown
a/duck/chiba/5/2006		AB537457	Research Team for Zoonotic Diseases; Kannondai 3-1-5, Tsukuba, Ibar	unknown
a/duck/chiba/8/06	EPI1139870		National Institute of Animal Health	National Institute of Animal Health

a/duck/chiba/8/2006		AB537460	Research Team for Zoonotic Diseases; Kannondai 3-1-5, Tsukuba, Iba	unknown
a/duck/chiba/6/2006		AB537458	Research Team for Zoonotic Diseases; Kannondai 3-1-5, Tsukuba, Iba	unknown
a/duck/chiba/43/2006		AB537467	Research Team for Zoonotic Diseases; Kannondai 3-1-5, Tsukuba, Iba	unknown
a/duck/chiba/9/2006		AB537461	Research Team for Zoonotic Diseases; Kannondai 3-1-5, Tsukuba, Iba	unknown
a/northern_pintail/alaska/78/2012		KY130779	U.S. Geological Survey, Alaska Science Center	unknown
a/green-winged_teal/alaska/ak19-540/2019	EPI2142844		unknown	unknown
a/northern_pintail/alaska/ak20-434/2020	EPI2143022		unknown	unknown
a/mallard_duck/south_korea/knu18-75/2018		PP746137	Wonkwang University School of Medicine, Infection Biology	unknown
a/northern_pintail/alaska/ugai15-7415/2015		KY131407	U.S. Geological Survey, Alaska Science Center	unknown
a/northern_pintail/alaska/ugai15-7417/2015		KY131415	U.S. Geological Survey, Alaska Science Center	unknown
a/emperor_goose/alaska/ugai15-8436/2015		MH569239	U.S. Geological Survey, Alaska Science Center	unknown
a/emperor_goose/alaska/ugai15-8432/2015		MH569223	U.S. Geological Survey, Alaska Science Center	unknown
a/emperor_goose/alaska/ugai15-8440/2015		MH569247	U.S. Geological Survey, Alaska Science Center	unknown
a/emperor_goose/alaska/ugai15-8448/2015		MH569279	U.S. Geological Survey, Alaska Science Center	unknown
a/emperor_goose/alaska/ugai15-8446/2015		MH569271	U.S. Geological Survey, Alaska Science Center	unknown
a/emperor_goose/alaska/ugai15-8444/2015		MH569255	U.S. Geological Survey, Alaska Science Center	unknown
a/emperor_goose/alaska/ugai15-8461/2015		MH569303	U.S. Geological Survey, Alaska Science Center	unknown
a/emperor_goose/alaska/ugai15-8463/2015		MH569311	U.S. Geological Survey, Alaska Science Center	unknown
a/emperor_goose/alaska/ugai15-8435/2015		MH569231	U.S. Geological Survey, Alaska Science Center	unknown
a/emperor_goose/alaska/ugai15-8445/2015		MH569263	U.S. Geological Survey, Alaska Science Center	unknown
a/glaucous-winged_gull/alaska/473/2012		KY130811	U.S. Geological Survey, Alaska Science Center	unknown
a/northern_pintail/alaska/483/2012		KY130819	U.S. Geological Survey, Alaska Science Center	unknown
a/duck/nagasaki/421017/2015	EPI855495		National Institute of Animal Health	National Institute of Animal Health
a/duck/hokkaido/w150/2015		MK978872	Hokkaido University, Faculty of Veterinary Medicine	unknown
a/northern_pintail/alaska/870/2014		KT338577	U.S. Geological Survey, Alaska Science Center	unknown
a/northern_pintail/alaska/861/2014		KT338561	U.S. Geological Survey, Alaska Science Center	unknown
a/northern_pintail/alaska/830/2014		KT338553	U.S. Geological Survey, Alaska Science Center	unknown
a/duck/gunma/1/2016	EPI867664		National Institute of Animal Health	unknown
a/duck/gunma/2/2016	EPI867648		National Institute of Animal Health	unknown
a/common_teal/kyoto/nies187/2021	EPI2158878		unknown	unknown
a/green-winged_teal_anas_crecca_/south_korea/knu2019-72/2019		MW391833	Zoonosis Research Center, School of Medicine, Wonkwang University	unknown
a/common_teal/sakhalin/od17/2019	EPI1638380		National Institute of Animal Health	Research Institute of Experimental and Clinical Me
a/common_teal/sakhalin/66c/2020	EPI1847527		National Institute of Animal Health	Research Institute of Experimental and Clinical Me
a/gadwall/amur_region/71b/2020	EPI1850013		National Institute of Animal Health	Research Institute of Experimental and Clinical Me
a/aquatic_bird/hong_kong/399/99		AJ427297	Chin P., Department of Microbiology, The University of Hong Kong, Uni	unknown
a/duck/zhejiang/d13/2013		KJ439848	School of Medicine, Zhejiang University, State Key Laboratory for Diag	unknown
a/duck/zhejiang/d17/2013		KJ439852	School of Medicine, Zhejiang University, State Key Laboratory for Diag	unknown
a/duck/zhejiang/d16/2013		KJ439851	School of Medicine, Zhejiang University, State Key Laboratory for Diag	unknown
a/duck/zhejiang/d11/2013		KJ439847	School of Medicine, Zhejiang University, State Key Laboratory for Diag	unknown
a/duck/zhejiang/d18/2013		KJ439853	School of Medicine, Zhejiang University, State Key Laboratory for Diag	unknown
a/duck/tsukuba/28/2006		AB537451	Research Team for Zoonotic Diseases; Kannondai 3-1-5, Tsukuba, Iba	unknown
a/northern_pintail/alaska/782/2012		KY130923	U.S. Geological Survey, Alaska Science Center	unknown
a/northern_pintail/alaska/52/2011		KY130536	U.S. Geological Survey, Alaska Science Center	unknown
a/green-winged_teal/alaska/ak20-372/2020	EPI2142852		unknown	unknown
a/green-winged_teal/alaska/ak18-wb1-066a/2018	EPI1772782		unknown	unknown
a/northern_pintail/alaska/ak19-130e_t1/2019	EPI2143155		unknown	unknown
a/mallard/alaska/ak19-199e_t1/2019	EPI2143129		unknown	unknown
a/american_green-winged_teal/alaska/137896/2009		KX714442	U.S. Geological Survey, Alaska Science Center	unknown
a/american_green-winged_teal/alaska/137916/2009		KX714450	U.S. Geological Survey, Alaska Science Center	unknown
a/northern_pintail/alaska/531/2011		KY130675	U.S. Geological Survey, Alaska Science Center	unknown
a/northern_pintail/alaska/649/2011		KY130707	U.S. Geological Survey, Alaska Science Center	unknown

a/green-winged_teal/alaska/816/2011		KY130739	U.S. Geological Survey, Alaska Science Center	unknown
a/northern_pintail/alaska/750/2011		KY130723	U.S. Geological Survey, Alaska Science Center	unknown
a/northern_pintail/alaska/562/2014		KT338460	U.S. Geological Survey, Alaska Science Center	unknown
a/northern_pintail/alaska/580/2014		KT338483	U.S. Geological Survey, Alaska Science Center	unknown
a/emperor_goose/alaska/ugai15-6737/2015		KX949460	U.S. Geological Survey, Alaska Science Center	unknown
a/emperor_goose/alaska/ugai15-6758/2015		KX949468	U.S. Geological Survey, Alaska Science Center	unknown
a/northern_pintail/alaska/ah0185234/2021		OL467092	National Veterinary Services Laboratories, Diagnostic Virology Labora	unknown
a/mallard/alaska/ah0185243/2021		OL467052	National Veterinary Services Laboratories, Diagnostic Virology Labora	unknown
a/mallard/alaska/ah0185252/2021		OL467068	National Veterinary Services Laboratories, Diagnostic Virology Labora	unknown
a/mallard/alberta/309/2022	EPI2258371		unknown	unknown
a/mallard/alberta/320/2022	EPI2258407		unknown	unknown
a/northern_pintail/alaska/ak19-534/2019	EPI2142998		unknown	unknown
a/common_teal/russia_primorje/177/2019	EPI1657075		National Institute of Animal Health	Research Institute of Experimental and Clinical Me
a/mallard/russia_primorje/182/2019	EPI1657009		National Institute of Animal Health	Research Institute of Experimental and Clinical Me
a/duck/vietnam/lbm12/2011		AB746482	Avian Zoonosis Research Center, Faculty of Agriculture; 4-101 Koyam	unknown
a/muscovy_duck/vietnam/lbm14/2011__h3n2_	EPI395171		unknown	unknown
a/long-tailed_duck/wisconsin/16os4632/2016		MG280318	St. Jude Center of Excellence for Influenza Research and Surveillance	unknown
a/duck/mongolia/oie-7799/2011		AB701298	Graduate School of Veterinary Medicine; kita-ku,kita18 nishi9, Sappo	unknown
a/duck/mongolia/496/2010	EPI1175845		unknown	unknown
a/duck/mongolia/444/2010		LC367362	Faculty of Veterinary Medicine; kita18 nishi9, kita-ku, Sapporo, Hokka	unknown
a/duck/mongolia/116/2011		LC339795	Faculty of Veterinary Medicine; kita18 nishi9, kita-ku, Sapporo, Hokka	unknown
a/duck/mongolia/71/2011	EPI1176466		unknown	unknown
a/duck/mongolia/29/2011	EPI1134037		unknown	unknown
a/duck/mongolia/569/2010		LC367426	Faculty of Veterinary Medicine; kita18 nishi9, kita-ku, Sapporo, Hokka	unknown
a/duck/mongolia/685/2010	EPI1176443		unknown	unknown
a/duck/mongolia/688/2010	EPI1176450		unknown	unknown
a/duck/mongolia/665/2010		LC367482	Faculty of Veterinary Medicine; kita18 nishi9, kita-ku, Sapporo, Hokka	unknown
a/anseriformes/anhui/l1/2014		MN148456	Guangdong Ocean University, Agriculture college	unknown
a/aquatic_bird/south_korea/sw006/2016		MG386182	Korea Research Institute of Bioscience and Biotechnology, Infectious	unknown
a/duck/mongolia/mn18-13/2018		MW487369	Zoonosis Research Center, School of Medicine, Department of Infecti	unknown
a/common_teal/russia_primorje/94/2019	EPI1657083		National Institute of Animal Health	Research Institute of Experimental and Clinical Me
a/common_teal/amur_region/31b/2019	EPI1638404		National Institute of Animal Health	Research Institute of Experimental and Clinical Me
a/wild_duck/shandong/w6271/2019	EPI2245832		Liaocheng University	College of Agronomy, Liaocheng University
a/wild_duck/shandong/w6293/2019	EPI2245880		Liaocheng University	College of Agronomy, Liaocheng University
a/wild_duck/shandong/w6279/2019	EPI2245856		Liaocheng University	College of Agronomy, Liaocheng University
a/wild_duck/shandong/w6277/2019	EPI2245848		Liaocheng University	College of Agronomy, Liaocheng University
a/wild_duck/shandong/w6275/2019	EPI2245840		Liaocheng University	College of Agronomy, Liaocheng University
a/wild_duck/shandong/w6268/2019	EPI2245824		Liaocheng University	College of Agronomy, Liaocheng University
a/duck/jiangsu/js1094/2019		OM527205	Yangzhou University, College of Veterinary Medicine	unknown
a/baikal_teal/xianghai/xh-15f/2012		KJ907551	Academy of Military Medical Sciences, Department of Virology Resea	unknown
a/duck/shanghai/74-1/2009		KU158894	Chinese Academy of Agricultural Sciences, Shanghai Veterinary Rese	unknown
a/duck/vietnam/lbm533/2013	EPI515135		unknown	unknown
a/duck/hunan/s1256/2012		CY146604	Harbin Veterinary Research Institute, Animal Influenza Laboratory of t	unknown
a/duck/hokkaido/221/2008	EPI1189823		unknown	unknown
a/pintail/mongolia/2-65/2007		JN029623	National Veterinary Research and Quarantine Service, Avian Disease	unknown
a/ruddy_shelduck/mongolia/2-79/2007		JN029642	National Veterinary Research and Quarantine Service, Avian Disease	unknown
a/whooper_swan/mongolia/1-17/2007		JN029557	National Veterinary Research and Quarantine Service, Avian Disease	unknown
a/duck/thailand/cu-11825c/2011		KJ161947	Chulalongkorn University, Veterinary Public Health	unknown
a/chicken/cambodia/kandaltbong/160602/2012/h3n6	EPI702679		Swedish Veterinary Agency (SVA)	National Veterinary Research Institute
a/duck/thailand/cu-11828c/2011	EPI506531		unknown	unknown
a/duck/thailand/cu-11831t/2011		KJ161949	Chulalongkorn University, Veterinary Public Health	unknown

a/duck/thailand/cu-11826c/2011		KJ161966	Chulalongkorn University, Veterinary Public Health	unknown
a/duck/thailand/cu-11824t/2011		KJ161963	Chulalongkorn University, Veterinary Public Health	unknown
a/duck/thailand/cu-11827t/2011		KJ161942	Chulalongkorn University, Veterinary Public Health	unknown
a/common_teal/sakhalin/110c/2020	EPI1847519		National Institute of Animal Health	Research Institute of Experimental and Clinical Me
a/common_teal/sakhalin/81c/2020	EPI1847535		National Institute of Animal Health	Research Institute of Experimental and Clinical Me
a/duck/guangdong/h31/2020	EPI1838454		South China Agricultural University	South China Agricultural University Veterinary Medi
a/northern_pintail/alaska/ak20-463/2020	EPI2143030		unknown	unknown
a/common_teal/sakhalin/od15/2019	EPI1638372		National Institute of Animal Health	Research Institute of Experimental and Clinical Me
a/common_teal/sakhalin/od18/2019	EPI1638388		National Institute of Animal Health	Research Institute of Experimental and Clinical Me
a/duck/hokkaido/w154/2017		MK592549	Hokkaido University, Faculty of Veterinary Medicine	unknown
a/duck/hokkaido/w151/2017		MK592541	Hokkaido University, Faculty of Veterinary Medicine	unknown
a/duck/hokkaido/w144/2017		MK592533	Hokkaido University, Faculty of Veterinary Medicine	unknown
a/wild_duck/shandong/w2021/2017	EPI2245808		Liaocheng University	College of Agronomy, Liaocheng University
a/wild_duck/shandong/w1765/2017	EPI2245766		Liaocheng University	College of Agronomy, Liaocheng University
a/wild_duck/shandong/w1766/2017	EPI2245774		Liaocheng University	College of Agronomy, Liaocheng University
a/duck/hokkaido/obf2/2018	EPI1776298		unknown	unknown
a/gadwall/amur_region/74b/2020	EPI1850021		National Institute of Animal Health	Research Institute of Experimental and Clinical Me
a/northern_pintail/russia_primorje/298/2019	EPI1657099		National Institute of Animal Health	Research Institute of Experimental and Clinical Me
a/mallard/alaska/ak18-wb2-185a/2018	EPI1772654		unknown	unknown
a/green-winged_teal/alaska/ak18-wb2-203a/2018	EPI1772798		unknown	unknown
a/mallard/alaska/ak18-wb1-048b/2018	EPI1772814		unknown	unknown
a/mallard/alaska/ak18-wb1-048a/2018	EPI1772806		unknown	unknown
a/northern_shoveler/mongolia/899v/2009		KF501066	School of Public Health, The University of Hong Kong	unknown
a/whooper_swan/mongolia/1-23/2007		JN029573	National Veterinary Research and Quarantine Service, Avian Disease	unknown
a/whooper_swan/mongolia/1-27/2007		JN029596	National Veterinary Research and Quarantine Service, Avian Disease	unknown
a/common_teal/mongolia/2271/2011		KF501098	School of Public Health, The University of Hong Kong	unknown
a/duck/mongolia/278/2011		LC349359	Faculty of Veterinary Medicine; kita18 nishi9, kita-ku, Sapporo, Hokka	unknown
a/duck/mongolia/279/2011		LC349367	Faculty of Veterinary Medicine; kita18 nishi9, kita-ku, Sapporo, Hokka	unknown
a/duck/mongolia/566/2018	EPI1510558		unknown	unknown
a/duck/mongolia/513/2018		MK978944	Hokkaido University, Faculty of Veterinary Medicine	unknown
a/duck/mongolia/734/2018	EPI1510622		unknown	unknown
a/duck/bangladesh/bdadai-3086/2019	EPI1932194		unknown	unknown
a/duck/bangladesh/3086/2019__h3n8_	EPI1888338		St. Jude Children's Research Hospital	Chattogram Veterinary and Animal Sciences Univer
a/duck/bangladesh/bdadai-3088/2019	EPI1932193		unknown	unknown
a/duck/bangladesh/3088/2019__h3n8_	EPI1888341		St. Jude Children's Research Hospital	Chattogram Veterinary and Animal Sciences Univer
a/duck/bangladesh/2204/2019__h3n8_	EPI1888008		St. Jude Children's Research Hospital	Chattogram Veterinary and Animal Sciences Univer
a/duck/bangladesh/bdadai-3147/2019	EPI2117004		unknown	unknown
a/duck/bangladesh/3147/2019__h3n8_	EPI1889101		St. Jude Children's Research Hospital	Chattogram Veterinary and Animal Sciences Univer
a/duck/bangladesh/2561/2019__h3n8_	EPI1888014		St. Jude Children's Research Hospital	Chattogram Veterinary and Animal Sciences Univer
a/duck/bangladesh/bdadai-3237/2019	EPI2117008		unknown	unknown
a/duck/bangladesh/3237/2019__h3n8_	EPI1889105		St. Jude Children's Research Hospital	Chattogram Veterinary and Animal Sciences Univer
a/duck/bangladesh/3089/2019__h3n8_	EPI1888342		St. Jude Children's Research Hospital	Chattogram Veterinary and Animal Sciences Univer
a/duck/bangladesh/bdadai-3089/2019	EPI1932197		unknown	unknown
a/duck/bangladesh/3084/2019__h3n8_	EPI1888336		St. Jude Children's Research Hospital	Chattogram Veterinary and Animal Sciences Univer
a/duck/bangladesh/bdadai-3094/2019	EPI1932204		unknown	unknown
a/duck/bangladesh/bdadai-3091/2019	EPI1932199		unknown	unknown
a/duck/bangladesh/3094/2019__h3n8_	EPI1889093		St. Jude Children's Research Hospital	Chattogram Veterinary and Animal Sciences Univer
a/duck/bangladesh/bdadai-3084/2019	EPI1933039		unknown	unknown
a/duck/bangladesh/3091/2019__h3n8_	EPI1889089		St. Jude Children's Research Hospital	Chattogram Veterinary and Animal Sciences Univer
a/chicken/bangladesh/2478/2019__h3n8_	EPI1888010		St. Jude Children's Research Hospital	Chattogram Veterinary and Animal Sciences Univer
a/duck/bangladesh/bdadai-3129/2019	EPI1932207		unknown	unknown

a/duck/bangladesh/3129/2019__h3n8_	EPI1889097		St. Jude Children's Research Hospital	Chattogram Veterinary and Animal Sciences Univer
a/duck/mongolia/575/2018		MK978960	Hokkaido University, Faculty of Veterinary Medicine	unknown
a/duck/guangxi/04.10_jx050/2015_mixed_	EPI667005		Institute of Microbiology, Chinese Academy of Sciences	unknown
a/chicken/ganzhou/gz157/2016		KY415634	National Institute for Communicable Disease Control and Prevention	unknown
a/chicken/ganzhou/gz43/2016		KY415635	National Institute for Communicable Disease Control and Prevention	unknown
a/mallard/netherlands/8/2013	EPI1530561		unknown	unknown
a/teal/chany/736/2008		CY100065	State Research Center of Virology and Biotechnology "Vector", Emerg	unknown
a/gadwall/altai/1326/2007		CY049804	State Research Center of Virology and Biotechnology "Vector"	unknown
a/gadwall/altai/1324/2007	EPI222055		unknown	unknown
a/gadwall/altai/1328/2007	EPI222079		unknown	unknown
a/duck/moscow/3806/2009	EPI1785223		unknown	unknown
a/mallard/sweden/599/2014		KY320426	Peter Doherty Institute for Infection and Immunity, WHO Collaboratin	unknown
a/northern_pintail/novosibirsk_region/3289k/2020	EPI1849973		National Institute of Animal Health	Research Institute of Experimental and Clinical Me
a/pintail/novosibirsk_region/519k/2018	EPI1353320		National Institute of Animal Health	Research Institute of Experimental and Clinical Me
a/anas_platyrhynchos/belgium/2499_0006/2021__mixed_	EPI2122991		Sciensano, Department of Animal Infectious Diseases	Sciensano - Animal Infectious Diseases
a/mallard/netherlands/29/2013	EPI1537027		unknown	unknown
a/anas_platyrhynchos/belgium/11294_004/2021	EPI1943018		Sciensano, Department of Animal Infectious Diseases	Sciensano - Animal Infectious Diseases
a/anas_platyrhynchos/belgium/11027_19/2017	EPI1774328		unknown	unknown
a/euraisan_coot/novosibirsk_region/3493k/2020	EPI1849869		National Institute of Animal Health	Research Institute of Experimental and Clinical Me
a/duck/mongolia/173/2015	EPI704323		unknown	unknown
a/duck/mongolia/179/2015	EPI704340		unknown	unknown
a/duck/bangladesh/20d677/2016	EPI1330330		Centers for Disease Control and Prevention	icddr,b International Centre for Diarrhoeal Disease
a/duck/bangladesh/20d678/2016	EPI1330337		Centers for Disease Control and Prevention	icddr,b International Centre for Diarrhoeal Disease
a/common_teal/chany/891/2018	EPI1333711		State Research Center of Virology and Biotechnology (VECTOR)	State Research Center of Virology and Biotechnolog
a/common_teal/chany/892/2018	EPI1333719		State Research Center of Virology and Biotechnology (VECTOR)	State Research Center of Virology and Biotechnolog
a/common_teal/chany/889/2018	EPI1333703		State Research Center of Virology and Biotechnology (VECTOR)	State Research Center of Virology and Biotechnolog
a/common_teal/chany/893/2018	EPI1333727		State Research Center of Virology and Biotechnology (VECTOR)	State Research Center of Virology and Biotechnolog
a/garganey/egypt/mb-d-1323c/2017		OQ793824	St. Jude Center of Excellence for Influenza Research and Surveillance	unknown
a/greylag_goose/north-kazakhstan/62/2019	EPI1790211		unknown	unknown
a/domestic_duck/iran/domestic_duck_375/2017		MW422771	Razi vaacine and serum research, Avian diseases	unknown
a/domestic_duck/iran/domestic_duck_379/2017		MW422885	Razi vaacine and serum research, Avian diseases	unknown
a/green_sandpiper/kurgan/1046/2018	EPI1440596		State Research Center of Virology and Biotechnology (VECTOR)	State Research Center of Virology and Biotechnolog
a/green_sandpiper/kurgan/1048/2018	EPI1440604		State Research Center of Virology and Biotechnology (VECTOR)	State Research Center of Virology and Biotechnolog
a/green_sandpiper/kurgan/1043/2018	EPI1440588		State Research Center of Virology and Biotechnology (VECTOR)	State Research Center of Virology and Biotechnolog
a/green_sandpiper/kurgan/1050/2018	EPI1440613		State Research Center of Virology and Biotechnology (VECTOR)	State Research Center of Virology and Biotechnolog
a/gadwall/omsk_region/129/2019	EPI1638540		National Institute of Animal Health	Research Institute of Experimental and Clinical Me
a/teal/dagestan/23d/2018	EPI1358860		National Institute of Animal Health	Research Institute of Experimental and Clinical Me
a/common_teal/novosibirsk_region/3556k/2020	EPI1850436		National Institute of Animal Health	Research Institute of Experimental and Clinical Me
a/coot/novosibirsk_region/563/2018	EPI1352494		National Institute of Animal Health	Research Institute of Experimental and Clinical Me
a/gadwall/novosibirsk_region/982k/2018	EPI1352462		National Institute of Animal Health	Research Institute of Experimental and Clinical Me
a/pintail/novosibirsk_region/518k/2018	EPI1352478		National Institute of Animal Health	Research Institute of Experimental and Clinical Me
a/common_teal/novosibirsk_region/3327k/2020	EPI1849837		National Institute of Animal Health	Research Institute of Experimental and Clinical Me
a/mallard/novosibirsk_region/3314k/2020	EPI1849893		National Institute of Animal Health	Research Institute of Experimental and Clinical Me
a/shoveler/novosibirsk_region/3465k/2020	EPI1849981		National Institute of Animal Health	Research Institute of Experimental and Clinical Me
a/anas_platyrhynchos/luxembourg/22154646/2022	EPI2283192		Luxembourg Institute of Health	Laboratoire de m√©decine v√©t√©rinaire de l'Etat
a/duck/moscow/6147/2022		PP754247	The Gamaleya National Research Centre for Epidemiology and Microb	unknown
a/wide_bird/ebinur_lake/47/2017		ON287065	South China Agricultural University, College of Veterinary Medicine	unknown
a/rook/novosibirsk/44-2v/2021	EPI1957929		State Research Center of Virology and Biotechnology (VECTOR)	State Research Center of Virology and Biotechnolog
a/sterna_paradisaea/fildes_peninsula__king_george_island/09/2023	EPI3349807		Central Research Institute of Epidemiology	Research Institute of Experimental and Clinical Me
a/duck/assam/ducl1512100011/2015		MT272413	ICAR-National Institute of High Security Animal Disease, Diagnostic I	unknown
a/duck/assam/duor1512100030/2015	EPI1889810		unknown	unknown

a/duck/assam/duor1512100004/2015		MT272421	ICAR-National Institute of High Security Animal Disease, Diagnostic I	unknown
a/common_teal/omsk_region/54/2019	EPI1638444		National Institute of Animal Health	Research Institute of Experimental and Clinical Me
a/anas_platyrhynchos/belgium/00330_0004/2023	EPI2495410		Sciensano, Department of Animal Infectious Diseases	Sciensano - Animal Infectious Diseases
a/anas_platyrhynchos/belgium/2214_0001/2021	EPI2122885		Sciensano, Department of Animal Infectious Diseases	Sciensano - Animal Infectious Diseases
a/anas_platyrhynchos/belgium/2987_0001/2021	EPI2122933		Sciensano, Department of Animal Infectious Diseases	Sciensano - Animal Infectious Diseases
a/duck/moscow/5881/2021		OP133624	The Gamaleya National Research Centre for Epidemiology and Microb	unknown
a/duck/chernogolovka/5897/2021		OP136011	The Gamaleya National Research Centre for Epidemiology and Microb	unknown
a/duck/chernogolovka/5908/2021		OP135951	The Gamaleya National Research Centre for Epidemiology and Microb	unknown
a/mallard/chany_lake/48/2018	EPI1353361		National Institute of Animal Health	Research Institute of Experimental and Clinical Me
a/teal/buryatia/63/2018	EPI1353336		National Institute of Animal Health	Research Institute of Experimental and Clinical Me
a/duck/mongolia/wku-38/2022	EPI2777224		unknown	unknown
a/duck/mongolia/wku-42/2022	EPI2777232		unknown	unknown
a/common_teal/chany_lake/38/2019	EPI1638428		National Institute of Animal Health	Research Institute of Experimental and Clinical Me
a/common_teal/novosibirsk_region/3515k/2020	EPI1850420		National Institute of Animal Health	Research Institute of Experimental and Clinical Me
a/common_teal/novosibirsk_region/3324k/2020	EPI1849829		National Institute of Animal Health	Research Institute of Experimental and Clinical Me
a/mallard/novosibirsk_region/3265k/2020	EPI1850029		National Institute of Animal Health	Research Institute of Experimental and Clinical Me
a/duck/nanchang/1681/1992		CY006016	St. Jude Children's Research Hospital, Hartwell Center for Bioinforma	unknown
a/mallard/new_zealand/449-84/2004	EPI297451		unknown	unknown
a/chestnut_teal/victoria/1/2004		CY094935	on behalf of JCVI/Australian Animal Healh Laboratory/NCBI, National	unknown
a/mallard/tasmania/06-0297-31/2006	EPI2143715		unknown	unknown
a/wild_duck/victoria/10-03507-019/2010		OL371215	CSIRO, Australian Centre for Disease Preparedness	unknown
a/ruddy_turnstone/tas/7048/2014	EPI1902328		unknown	unknown
a/ruddy_turnstone/tas/7040/2014	EPI1902321		unknown	unknown
a/ruddy_turnstone/tas/7049/2014	EPI1902334		unknown	unknown
a/wild_waterfowl/south_australia/15-5472990-54/2015	EPI2145843		unknown	unknown
a/pacific_black_duck/victoria/9718/2016		OL370551	CSIRO, Australian Centre for Disease Preparedness	unknown
a/pacific_black_duck/victoria/10582/2017		OL370495	CSIRO, Australian Centre for Disease Preparedness	unknown
a/pink-eared_duck/victoria/10826/2017	EPI2144435		unknown	unknown
a/wild_waterfowl/western_australia/as16-3326-127/2016	EPI2145931		unknown	unknown
a/wild_waterfowl/western_australia/as16-3326-174/2016	EPI2145939		unknown	unknown
a/wild_duck/tasmania/16-0910-19/2016		OL371135	CSIRO, Australian Centre for Disease Preparedness	unknown
a/wild_duck/tasmania/16-0910-8/2016		OL371143	CSIRO, Australian Centre for Disease Preparedness	unknown
a/grey_teal/victoria/10834/2017	EPI2144395		unknown	unknown
a/ruddy_turnstone/king_island/11986/2017	EPI2144491		unknown	unknown
a/ruddy_turnstone/king_island/11988/2017	EPI2144499		unknown	unknown
a/ruddy_turnstone/king_island/11990/2017		OL370735	CSIRO, Australian Centre for Disease Preparedness	unknown
a/ruddy_turnstone/king_island/12012/2017	EPI2144523		unknown	unknown
a/ruddy_turnstone/king_island/11991/2017	EPI2144515		unknown	unknown
a/wild_waterfowl/queensland/p19-01624-26/2019	EPI2144085		unknown	unknown
a/wild_waterfowl/queensland/p19-01624-24/2019	EPI2145795		unknown	unknown
a/wild_waterfowl/queensland/p19-01624-27/2019	EPI2145803		unknown	unknown
a/wild_waterfowl/queensland/p19-01624-17/2019	EPI2145763		unknown	unknown
a/wild_waterfowl/queensland/p19-01624-19/2019	EPI2145779		unknown	unknown
a/wild_waterfowl/queensland/p19-01624-28/2019	EPI2145811		unknown	unknown
a/wild_waterfowl/queensland/p19-01624-18/2019	EPI2145771		unknown	unknown
a/wild_waterfowl/queensland/p19-01624-22/2019	EPI2145787		unknown	unknown
a/duck/guangxi/n42/2009	EPI321533		unknown	unknown
a/duck/guangxi/69/2009		HQ647010	Guangxi Center for Animal Disease Prevention and Control	unknown
a/goose/guangxi/139g20/2013	EPI1006583		unknown	unknown
a/chicken/guangxi/125c8/2012	EPI1006551		unknown	unknown
a/pigeon/guangxi/128p9/2012	EPI1006567		unknown	unknown

a/chicken/guangxi/135c10/2013	EPI1006559		unknown	unknown
a/duck/guangxi/135d20/2013	EPI1006575		unknown	unknown
a/wild_chicken/anhui/f70/2015		MN148504	Guangdong Ocean University, Agriculture college	unknown
a/duck/china/influenza_a_virus/2019	EPI1930856		unknown	unknown
a/duck/jiangsu/js1264/2020		OM527213	Yangzhou University, College of Veterinary Medicine	unknown
a/duck/jiangsu/js0451/2021		OM527189	Yangzhou University, College of Veterinary Medicine	unknown
a/duck/shandong/sd0220/2021		OM527221	Yangzhou University, College of Veterinary Medicine	unknown
a/duck/guangxi/3846/2020	EPI2594475		unknown	unknown
a/duck/vietnam/hn5681/2019		MW934771	Center for Research on Influenza Pathogenesis (CRIP), CEIRS Data Pr	unknown
a/duck/vietnam/hn5687/2019		MW935164	Center for Research on Influenza Pathogenesis (CRIP), CEIRS Data Pr	unknown
a/duck/vietnam/hn5690/2019		MW935208	Center for Research on Influenza Pathogenesis (CRIP), CEIRS Data Pr	unknown
a/duck/vietnam/hn5686/2019		MW935365	Center for Research on Influenza Pathogenesis (CRIP), CEIRS Data Pr	unknown
a/duck/vietnam/hn5689/2019		MW935335	Center for Research on Influenza Pathogenesis (CRIP), CEIRS Data Pr	unknown
a/duck/vietnam/hn5685/2019		MW935200	Center for Research on Influenza Pathogenesis (CRIP), CEIRS Data Pr	unknown
a/duck/vietnam/hn5688/2019		MW935292	Center for Research on Influenza Pathogenesis (CRIP), CEIRS Data Pr	unknown
a/duck/vietnam/hn5684/2019		MW934700	Center for Research on Influenza Pathogenesis (CRIP), CEIRS Data Pr	unknown
a/duck/vietnam/lbm446/2013		AB916643	Avian Zoonosis Research Center, Faculty of Agriculture; 4-101 Koyam	unknown
a/muscovy_duck/vietnam/lbm348/2013	EPI596408		unknown	unknown
a/duck/vietnam/lbm798/2014	EPI590045		unknown	unknown
a/duck/vietnam/hu5-481/2016		LC427491	Faculty of Veterinary Medicine; Kita18 Nishi9, Kita-ku, Sapporo, Hokk	unknown
a/duck/nha_trang/169/2016		LC496160	Avian Zoonosis Research Center, Faculty of Agriculture; 4-101, Koyam	unknown
a/duck/vietnam/lbm836/2015		LC496120	Avian Zoonosis Research Center, Faculty of Agriculture; 4-101, Koyam	unknown
a/duck/vietnam/1501/2014	EPI585381		unknown	unknown
a/duck/vietnam/hu1-1501/2014		LC041332	Microbio laboratory; Kita 18 Nishi 9, Kitaku, Sappaoro, Hokkaido 060-	unknown
a/duck/zhejiang/4812/2013		KF357821	First Affiliated Hospital, College of Medicine, Zhejiang University, Stat	unknown
a/wild_birds/hubei/137/2014	EPI1785779		unknown	unknown
a/duck/jiangshu/yz916/2016		MG021165	Yangzhou university, School of Veterinary Medicine, Yangzhou Univers	unknown
a/duck/wuxi/7275/2016	EPI1055305		Beijing Institute of Microbiology and Epidemiology	Beijing Institute of Microbiology and Epidemiology
a/goose/wuxi/7276/2016	EPI1055313		Beijing Institute of Microbiology and Epidemiology	Beijing Institute of Microbiology and Epidemiology
a/duck/hunan/146/2014		KX121254	Lanzhou Veterinary Research Institute	unknown
a/duck/jiangsu/ad/2016	EPI1536929		unknown	unknown
a/duck/anhui/a1459/2014		KP765887	China Animal Health and Epidemiology Center, Laboratory of Avian Di	unknown
a/duck/anhui/a1831/2014	EPI708150		unknown	unknown
a/duck/anhui/a1838/2014		KP765930	China Animal Health and Epidemiology Center, Laboratory of Avian Di	unknown
a/duck/anhui/a1781/2014		KP765902	China Animal Health and Epidemiology Center, Laboratory of Avian Di	unknown
a/duck/anhui/a1710/2014		KP765901	China Animal Health and Epidemiology Center, Laboratory of Avian Di	unknown
a/duck/anhui/a1649/2014		KP765898	China Animal Health and Epidemiology Center, Laboratory of Avian Di	unknown
a/duck/anhui/a1650/2014		KP765899	China Animal Health and Epidemiology Center, Laboratory of Avian Di	unknown
a/duck/anhui/a1651/2014		KP765900	China Animal Health and Epidemiology Center, Laboratory of Avian Di	unknown
a/duck/jiangsu/j2186/2014		KP767558	China Animal Health and Epidemiology Center, Laboratory of Avian Di	unknown
a/duck/jiangsu/j2190/2014		KP767559	China Animal Health and Epidemiology Center, Laboratory of Avian Di	unknown
a/duck/jiangsu/j2291/2014		KP767576	China Animal Health and Epidemiology Center, Laboratory of Avian Di	unknown
a/duck/jiangsu/j2276/2014		KP767565	China Animal Health and Epidemiology Center, Laboratory of Avian Di	unknown
a/duck/jiangsu/j2195/2014		KP767560	China Animal Health and Epidemiology Center, Laboratory of Avian Di	unknown
a/duck/jiangsu/j2414/2014		KP767586	China Animal Health and Epidemiology Center, Laboratory of Avian Di	unknown
a/duck/jiangsu/j2196/2014		KP767561	China Animal Health and Epidemiology Center, Laboratory of Avian Di	unknown
a/duck/jiangsu/j2202/2014		KP767563	China Animal Health and Epidemiology Center, Laboratory of Avian Di	unknown
a/duck/jiangsu/j2277/2014	EPI705514		unknown	unknown
a/duck/jiangsu/j2411/2014		KP767585	China Animal Health and Epidemiology Center, Laboratory of Avian Di	unknown
a/duck/jiangsu/j2280/2014		KP767568	China Animal Health and Epidemiology Center, Laboratory of Avian Di	unknown
a/duck/jiangsu/j2293/2014		KP767577	China Animal Health and Epidemiology Center, Laboratory of Avian Di	unknown

a/duck/jiangsu/j2282/2014		KP767570	China Animal Health and Epidemiology Center, Laboratory of Avian Di	unknown
a/duck/jiangsu/j2410/2014		KP767584	China Animal Health and Epidemiology Center, Laboratory of Avian Di	unknown
a/duck/jiangsu/j2274/2014		KP767564	China Animal Health and Epidemiology Center, Laboratory of Avian Di	unknown
a/northern_pintail/alaska/44340-221/2007		GU143853	USGS - Alaska Science Center	unknown
a/american_green-winged_teal/alaska/98313/2008		KX714362	U.S. Geological Survey, Alaska Science Center	unknown
a/northern_pintail/alaska/44155-158/2006		EU557492	USGS Alaska Science Center	unknown
a/northern_pintail/alaska/44228-175/2006		EU557504	USGS Alaska Science Center	unknown
a/northern_pintail/alaska/44228-129/2006		EU557502	USGS Alaska Science Center	unknown
a/mallard/oregon/44221-105/2006	EPI290548		unknown	unknown
a/double-crested_cormorant/california/20119-001/2007	EPI290615		unknown	unknown
a/spectacled_eider/alaska/44173-055/2006		JX080781	U.S. Geological Survey, Alaska Science Center	unknown
a/northern_pintail/alaska/44421-431/2008		GU143862	USGS - Alaska Science Center	unknown
a/northern_pintail/alaska/44421-446/2008		GU143866	USGS - Alaska Science Center	unknown
a/mallard/alberta/398/2017	EPI1299844		unknown	unknown
a/american_wigeon/utah/d1615126/2016	EPI1549552		unknown	unknown
a/mallard/alberta/203/2015	EPI1072152		unknown	unknown
a/american_black_duck/alberta/96/2016		MF613845	St. Jude Center of Excellence for Influenza Research and Surveillance	unknown
a/mallard/alberta/66/2016		MF046387	St. Jude Center of Excellence for Influenza Research and Surveillance	unknown
a/american_widgeon/interior_alaska/1/2007	EPI221444		unknown	unknown
a/green-winged_teal/interior_alaska/6mp1077/2006		CY078539	on behalf of JCVI/University of Alaska Fairbanks/NCBI, National Cente	unknown
a/mallard/interior_alaska/6mp0952/2006	EPI298674		unknown	unknown
a/green-winged_teal/interior_alaska/6mp0909/2006	EPI298538		unknown	unknown
a/mallard/interior_alaska/6mp0124/2006		CY078875	on behalf of JCVI/University of Alaska Fairbanks/NCBI, National Cente	unknown
a/northern_pintail/alaska/956/2012		KY130961	U.S. Geological Survey, Alaska Science Center	unknown
a/green-winged_teal/alaska/292/2011		KY130616	U.S. Geological Survey, Alaska Science Center	unknown
a/northern_pintail/alaska/779/2012		KY130907	U.S. Geological Survey, Alaska Science Center	unknown
a/northern_pintail/alaska/605/2012	EPI966589		unknown	unknown
a/emperor_goose/alaska/18/2012		KY130771	U.S. Geological Survey, Alaska Science Center	unknown
a/emperor_goose/alaska/279/2012		KY130787	U.S. Geological Survey, Alaska Science Center	unknown
a/green-winged_teal/alaska/781/2012		KY130915	U.S. Geological Survey, Alaska Science Center	unknown
a/northern_pintail/alaska/887/2012		KY130953	U.S. Geological Survey, Alaska Science Center	unknown
a/green-winged_teal/alaska/239/2013		KY130977	U.S. Geological Survey, Alaska Science Center	unknown
a/northern_pintail/alaska/619/2013		KY131104	U.S. Geological Survey, Alaska Science Center	unknown
a/northern_pintail/alaska/404/2013		KY131025	U.S. Geological Survey, Alaska Science Center	unknown
a/mallard/alaska/327/2013		KY130993	U.S. Geological Survey, Alaska Science Center	unknown
a/northern_pintail/alaska/362/2013		KY131001	U.S. Geological Survey, Alaska Science Center	unknown
a/northern_pintail/alaska/778/2012		KY130899	U.S. Geological Survey, Alaska Science Center	unknown
a/northern_pintail/alaska/500/2012		KY130835	U.S. Geological Survey, Alaska Science Center	unknown
a/northern_pintail/alaska/687/2012		KY130875	U.S. Geological Survey, Alaska Science Center	unknown
a/green-winged_teal/alaska/ak20-528/2020	EPI2142876		unknown	unknown
a/northern_pintail/alaska/ugai16-5343/2016	EPI1903886		unknown	unknown
a/american_green-winged_teal/alaska/306/2014		KT338349	U.S. Geological Survey, Alaska Science Center	unknown
a/american_green-winged_teal/alaska/478/2014		KT338420	U.S. Geological Survey, Alaska Science Center	unknown
a/american_green-winged_teal/alaska/524/2014		KT338452	U.S. Geological Survey, Alaska Science Center	unknown
a/northern_pintail/alaska/523/2014		KT338444	U.S. Geological Survey, Alaska Science Center	unknown
a/american_green-winged_teal/alaska/19tl00145/2019	EPI1894633		unknown	unknown
a/northern_pintail/alaska/19tl00162/2019	EPI1894615		unknown	unknown
a/mallard/alaska/nd0006301/2013		KM374026	National Veterinary Services Laboratories (NVSL), USDA, Diagnostic V	unknown
a/northern_pintail/alaska/ugai15-7399/2015		KY131392	U.S. Geological Survey, Alaska Science Center	unknown
a/green-winged_teal/alaska/ugai16-4610/2016	EPI1903824		unknown	unknown
a/northern_pintail/alaska/ugai16-4606/2016	EPI1903832		unknown	unknown

a/mallard/southcentral_alaska/12ml00982/2014	EPI762247		unknown	unknown
a/mallard/southcentral_alaska/12ml01001_aaf1/2014		CY194205	on behalf of JCVI/Massachusetts Institute of Technology/NCBI, Natior	unknown
a/mallard/southcentral_alaska/12ml01012/2014	EPI762310		unknown	unknown
a/mallard/southcentral_alaska/12ml00993/2014	EPI762282		unknown	unknown
a/mallard/southcentral_alaska/12ml00986_aaf1/2014		CY194173	on behalf of JCVI/Massachusetts Institute of Technology/NCBI, Natior	unknown
a/mallard/southcentral_alaska/12ml00997_aaf1/2014		CY194197	on behalf of JCVI/Massachusetts Institute of Technology/NCBI, Natior	unknown
a/mallard/southcentral_alaska/12ml00974/2014	EPI762255		unknown	unknown
a/mallard/southcentral_alaska/12ml01011/2014	EPI762274		unknown	unknown
a/duck/guangdong/455/2000		KF258949	The University of Hong Kong, State Key Laboratory of Emerging Infectio	unknown
a/duck/zhejiang/6d4/2013		KJ439849	School of Medicine, Zhejiang University, State Key Laboratory for Diag	unknown
a/duck/zhejiang/6d7/2013		KJ439850	School of Medicine, Zhejiang University, State Key Laboratory for Diag	unknown
a/duck/guangdong/03.26_dgcp072-p/2015_h3n8_	EPI659927		Institute of Microbiology, Chinese Academy of Sciences	unknown
a/duck/guangdong/04.22_dgcp075-p/2015_mixed_	EPI666749		Institute of Microbiology, Chinese Academy of Sciences	unknown
a/duck/guangdong/04.22_dgcp074-p/2015_h3n2_	EPI660919		Institute of Microbiology, Chinese Academy of Sciences	unknown
a/duck/guangdong/04.22_dgcp078-p/2015_mixed_	EPI666365		Institute of Microbiology, Chinese Academy of Sciences	unknown
a/duck/guangdong/04.22_dgcp064-p/2015_mixed_	EPI666133		Institute of Microbiology, Chinese Academy of Sciences	unknown
a/chicken/guangxi/2117/2010		HQ874606	Guangxi Center for Animal Disease Prevention and Control	unknown
a/duck/guangxi/015d2/2009	EPI1006519		unknown	unknown
a/white-backed_munia/hong_kong/4519/2009	EPI254282		unknown	unknown
a/chicken/guangxi/015c10/2009	EPI1006511		unknown	unknown
a/duck/guangxi/057d6/2010	EPI1006527		unknown	unknown
a/chicken/guangxi/073c2/2010	EPI1006535		unknown	unknown
a/duck/guangxi/112d4/2012	EPI1006543		unknown	unknown
a/duck/shanghai/c84/2009	EPI398851		unknown	unknown
a/mallard/jiangxi/1-4/2010		CY098262	Wuhan Institute Of Virology, Chinese Academy Of Sciences, Virus Res	unknown
a/mallard/jiangxi/1-19/2010	EPI545904		unknown	unknown
a/mallard/jiangxi/2-5/2010		CY098270	Wuhan Institute Of Virology, Chinese Academy Of Sciences, Virus Res	unknown
a/duck/guangdong/w12/2011		JX175253	South China Agricultural University, College of Veterinary Medicine	unknown
a/swine/guangdong/l21/2011		JX096504	College of Veterinary Medicine, South China Agricultural University	unknown
a/duck/guangdong/g1529/2014		KP765949	China Animal Health and Epidemiology Center, Laboratory of Avian Di	unknown
a/duck/guangdong/04.22_dgcp083-p/2015_h3n8_	EPI660359		Institute of Microbiology, Chinese Academy of Sciences	unknown
a/duck/quang_ninh/220/2014	EPI590034		unknown	unknown
a/duck/guangdong/04.22_dgcp068-p/2015_mixed_	EPI666893		Institute of Microbiology, Chinese Academy of Sciences	unknown
a/duck/guangdong/04.22_dgcp070-o/2015_mixed_	EPI666277		Institute of Microbiology, Chinese Academy of Sciences	unknown
a/duck/guangdong/g3077/2014		KP767629	China Animal Health and Epidemiology Center, Laboratory of Avian Di	unknown
a/duck/zhejiang/4625/2013		KF357819	First Affiliated Hospital, College of Medicine, Zhejiang University, Stat	unknown
a/duck/zhejiang/4613/2013		KF357818	First Affiliated Hospital, College of Medicine, Zhejiang University, Stat	unknown
a/duck/zhejiang/4637/2013		KF357820	First Affiliated Hospital, College of Medicine, Zhejiang University, Stat	unknown
a/duck/hunan/121/2014		KX121214	Lanzhou Veterinary Research Institute	unknown
a/chicken/vietnam/g14/2008		AB593455	Faculty of Life Sciences; Motoyama, Kamigamo, Kita-Ku, Kyoto, Kyoto	unknown
a/duck/fujian/sd063/2017		MG204059	Xiamen Center for Animal Disease Control and Prevention, Veterinary	unknown
a/duck/hunan/161/2015		KX121262	Lanzhou Veterinary Research Institute	unknown
a/duck/foshan/11/2019	EPI2376562		unknown	unknown
a/goose/guangdong/g630/2019/h3n2	EPI2088431		South China Agricultural University	South China Agricultural University
a/duck/guangdong/h34/2020/h3n2	EPI2088439		South China Agricultural University	South China Agricultural University
a/chicken/sichuan/h157/2020/h3n2	EPI2088471		South China Agricultural University	South China Agricultural University
a/duck/guangdong/h140/2020/h3n2	EPI2088447		South China Agricultural University	South China Agricultural University
a/duck/guangxi/h151/2020/h3n2	EPI2088463		South China Agricultural University	South China Agricultural University
a/duck/guangdong/h144/2020/h3n2	EPI2088455		South China Agricultural University	South China Agricultural University
a/duck/hunan/7/2015		KX121270	Lanzhou Veterinary Research Institute	unknown
a/duck/guangdong/f138/2017	EPI1328404		South China Agricultural University	unknown

a/duck/hubei/zysyf18/2015		KY415605	National Institute for Communicable Disease Control and Prevention	unknown
a/duck/hubei/zysyf12/2015		KY415604	National Institute for Communicable Disease Control and Prevention	unknown
a/duck/hubei/zysyf4/2015	EPI942059		unknown	unknown
a/duck/hubei/zysyf21/2015		KY415612	National Institute for Communicable Disease Control and Prevention	unknown
a/duck/hubei/zysyf2/2015		KY415614	National Institute for Communicable Disease Control and Prevention	unknown
a/duck/hubei/zysyf9/2015		KY415613	National Institute for Communicable Disease Control and Prevention	unknown
a/duck/guangdong/04.16_szlgwl012/2015_mixed_	EPI667269		Institute of Microbiology, Chinese Academy of Sciences	unknown
a/chicken/guangdong/g152/2019/h3n2	EPI2088406		South China Agricultural University	South China Agricultural University
a/chicken/guangdong/g155/2019/h3n2	EPI2088415		South China Agricultural University	South China Agricultural University
a/chicken/guangxi/165c7/2014	EPI1006591		unknown	unknown
a/duck/guangxi/175d12/2014	EPI615106		unknown	unknown
a/duck/guangdong/f352/2018	EPI1328406		South China Agricultural University	unknown
a/chicken/guangdong/f117/2018	EPI1489636		South China Agricultural University	South China Agricultural University Veterinary Medi
a/duck/guangdong/f1172/2018	EPI1489625		unknown	unknown
a/duck/china/322d22/2018		MN443576	Guangxi Veterinary Rerearch Institute, Guangxi Key Laboratory of Vete	unknown
a/duck/china/503d32/2022		PP474703	Guangxi Veterinary Rerearch Institute, Guangxi Key Laboratory of Vete	unknown
a/duck/china/400d17/2019		MW368487	Guangxi Veterinary Rerearch Institute, Guangxi Key Laboratory of Vete	unknown
a/duck/china/532d55/2023		PP474946	Guangxi Veterinary Rerearch Institute, Guangxi Key Laboratory of Vete	unknown
a/duck/china/401d23/2019		MW368618	Guangxi Veterinary Rerearch Institute, Guangxi Key Laboratory of Vete	unknown
a/duck/china/403d49/2020		MW368609	Guangxi Veterinary Rerearch Institute, Guangxi Key Laboratory of Vete	unknown
a/duck/guangxi/4130/2020	EPI2594498		unknown	unknown
a/chicken/jiangxi/11740/2022	EPI2595693		unknown	unknown
a/chicken/jiangxi/10541/2022	EPI2594804		unknown	unknown
a/chicken/jiangxi/37324/2021	EPI2595567		unknown	unknown
a/chicken/jiangxi/6979/2022	EPI2595620		unknown	unknown
a/chicken/jiangxi/11774/2022	EPI2595863		unknown	unknown
a/chicken/jiangxi/37317/2021	EPI2595519		unknown	unknown
a/chicken/jiangxi/3236/2022	EPI2595887		unknown	unknown
a/chicken/jiangxi/772/2022	EPI2594937		unknown	unknown
a/chicken/jiangxi/8027/2022	EPI2595789		unknown	unknown
a/chicken/shantou/6641/2021		OQ293237	The University of Hong Kong, School of Public Health	unknown
a/chicken/guangdong/343/2022	EPI2595005		unknown	unknown
a/silkie_chicken/shantou/237/2022		OQ293573	The University of Hong Kong, School of Public Health	unknown
a/silkie_chicken/shantou/345/2022		OQ293589	The University of Hong Kong, School of Public Health	unknown
a/chicken/guangdong/345/2022	EPI2595038		unknown	unknown
a/chicken/guangdong/6395/2021	EPI2594509		unknown	unknown
a/silkie_chicken/shantou/6747/2021		OQ293829	The University of Hong Kong, School of Public Health	unknown
a/chicken/shantou/6650/2021		OQ293245	The University of Hong Kong, School of Public Health	unknown
a/chicken/guangdong/6597/2021	EPI2594387		unknown	unknown
a/chicken/guangdong/6675/2021	EPI2594219		unknown	unknown
a/chicken/shantou/55/2022		OQ293021	The University of Hong Kong, School of Public Health	unknown
a/chicken/shantou/42/2022		OQ292781	The University of Hong Kong, School of Public Health	unknown
a/chicken/dongguan/734/2022		OQ291813	The University of Hong Kong, School of Public Health	unknown
a/chicken/guangdong/2703/2021	EPI2594732		unknown	unknown
a/chicken/huizhou/104/2022		OQ291989	The University of Hong Kong, School of Public Health	unknown
a/chicken/guangdong/2728/2021	EPI2594756		unknown	unknown
a/chicken/guangdong/677/2022	EPI2594902		unknown	unknown
a/chicken/huizhou/567/2022		OQ292229	The University of Hong Kong, School of Public Health	unknown
a/silkie_chicken/huizhou/256/2022		OQ293517	The University of Hong Kong, School of Public Health	unknown
a/chicken/guangdong/261/2022	EPI2594834		unknown	unknown
a/silkie_chicken/huizhou/261/2022		OQ293525	The University of Hong Kong, School of Public Health	unknown

a/chicken/dongguan/78/2022		OQ291829	The University of Hong Kong, School of Public Health	unknown
a/chicken/guangdong/116/2022	EPI2594594		unknown	unknown
a/silkie_chicken/dongguan/111/2022		OQ293437	The University of Hong Kong, School of Public Health	unknown
a/chicken/guangdong/3829/2021	EPI2594658		unknown	unknown
a/chicken/hong_kong/21-17040/2021	EPI2200708		unknown	unknown
a/chicken/hong_kong/21-17632/2021	EPI2200716		unknown	unknown
a/chicken/dongguan/3560/2021		OQ291653	The University of Hong Kong, School of Public Health	unknown
a/chicken/guangdong/404/2022	EPI2595443		unknown	unknown
a/chicken/dongguan/370/2022		OQ291669	The University of Hong Kong, School of Public Health	unknown
a/chicken/guangdong/370/2022	EPI2594674		unknown	unknown
a/chicken/shantou/5386/2021		OQ293013	The University of Hong Kong, School of Public Health	unknown
a/chicken/shantou/5632/2021		OQ293037	The University of Hong Kong, School of Public Health	unknown
a/chicken/guangdong/4181/2021	EPI2594183		unknown	unknown
a/silkie_chicken/shantou/4623/2021		OQ293677	The University of Hong Kong, School of Public Health	unknown
a/silkie_chicken/shantou/4624/2021		OQ293685	The University of Hong Kong, School of Public Health	unknown
a/chicken/guangdong/6414/2021	EPI2594483		unknown	unknown
a/chicken/shantou/6601/2021		OQ293221	The University of Hong Kong, School of Public Health	unknown
a/chicken/guangdong/6442/2021	EPI2594459		unknown	unknown
a/chicken/shantou/4541/2021		OQ292861	The University of Hong Kong, School of Public Health	unknown
a/chicken/guangdong/5811/2021	EPI2593849		unknown	unknown
a/chicken/shantou/4367/2021		OQ292789	The University of Hong Kong, School of Public Health	unknown
a/chicken/shantou/4425/2021		OQ292821	The University of Hong Kong, School of Public Health	unknown
a/silkie_chicken/shantou/4625/2021		OQ293693	The University of Hong Kong, School of Public Health	unknown
a/chicken/guangdong/4771/2021	EPI2594094		unknown	unknown
a/chicken/shantou/4699/2021		OQ292925	The University of Hong Kong, School of Public Health	unknown
a/chicken/shantou/4683/2021		OQ292893	The University of Hong Kong, School of Public Health	unknown
a/chicken/shantou/6045/2021		OQ293061	The University of Hong Kong, School of Public Health	unknown
a/chicken/shantou/6063/2021		OQ293069	The University of Hong Kong, School of Public Health	unknown
a/changsha/1000/2022	EPI2035832		Changsha Disease Prevention and Control Center	Changsha Disease Prevention and Control Center
a/chicken/dongguan/584/2022		OQ291805	The University of Hong Kong, School of Public Health	unknown
a/guangdong/zs-23sf005/2023	EPI2508604		Guangdong Provincial Center for Disease Control and Prevention	Guangdong Provincial Center for Disease Control a
a/chicken/shantou/481/2022		OQ292957	The University of Hong Kong, School of Public Health	unknown
a/chicken/anhui/fe12/2022	EPI2047366		China Agricultural University	China Agricultural University
a/henan/4-10/2022	EPI2024815		Henan Center for Disease Control and Prevention, China	Henan provincial center for disease control and pre
a/chicken/jiangsu/a3123/2022	EPI2047609		China Agricultural University	China Agricultural University
a/chicken/china/gt2125/2023		PP077078	henan agricultural university, 517	unknown
a/chicken/china/hunau-gt2125/2023	EPI2896244		Henan Agricultural University	Henan Agricultural University
a/chicken/china/hn0120/2023		PP838576	Henan Agricultural University, Bei517	unknown
a/chicken/henan_shangqiu/sq2049/2023		PP758466	Henan Agricultural University, School of Veterinary Medicine	unknown
a/duck/vietnam/oie-2382/2009	EPI243472		unknown	unknown
a/duck/cambodia/537w15m1/2013	EPI700301		Institut Pasteur du Cambodia	Institut Pasteur du Cambodia
a/duck/cambodia/849w25m3/2013	EPI700300		Institut Pasteur du Cambodia	Institut Pasteur du Cambodia
a/duck/east_java/av39/2013	EPI1215591		Kobe University Graduate School of Medicine	Institute of Tropical Disease, Universitas Airlangga
a/duck/east_java/av689/2016	EPI1640796		Kobe University Graduate School of Medicine	Institute of Tropical Disease, Universitas Airlangga
a/duck/east_java/av694/2016	EPI1640804		Kobe University Graduate School of Medicine	Institute of Tropical Disease, Universitas Airlangga
a/duck/east_java/spg79/2018	EPI1494358		Kobe University Graduate School of Medicine	Institute of Tropical Disease, Universitas Airlangga
a/duck/east_java/spg258/2019	EPI1647366		Kobe University Graduate School of Medicine	Institute of Tropical Disease, Universitas Airlangga
a/duck/east_java/av771/2016	EPI1640925		Kobe University Graduate School of Medicine	Institute of Tropical Disease, Universitas Airlangga
a/duck/east_java/av995/2016	EPI1640981		Kobe University Graduate School of Medicine	Institute of Tropical Disease, Universitas Airlangga
a/duck/east_java/spg194/2018	EPI1494326		Kobe University Graduate School of Medicine	Institute of Tropical Disease, Universitas Airlangga
a/duck/east_java/spg63/2017	EPI1647358		Kobe University Graduate School of Medicine	Institute of Tropical Disease, Universitas Airlangga

a/duck/east_java/spg82/2018	EPI1494318		Kobe University Graduate School of Medicine	Institute of Tropical Disease, Universitas Airlangga
a/duck/east_java/spg75/2018	EPI1494334		Kobe University Graduate School of Medicine	Institute of Tropical Disease, Universitas Airlangga
a/duck/east_java/spg78/2018	EPI1494190		Kobe University Graduate School of Medicine	Institute of Tropical Disease, Universitas Airlangga
a/duck/shanghai/sh1/2013		KM222556	Shanghai Animal Disease Control Center	unknown
a/duck/shanghai/sh2/2013		KM222564	Shanghai Animal Disease Control Center	unknown
a/duck/zhejiang/5/2011		JX051229	Zhejiang Academy of Medical Sciences, Institute of Bioengineering	unknown
a/chicken/guangdong/g1863/2014		KP765881	China Animal Health and Epidemiology Center, Laboratory of Avian Di	unknown
a/duck/anhui/d293/2014		KT022355	Key Laboratory of Zoonosis of Ministry of Agriculture, College of Veteri	unknown
a/duck/zhejiang/d1-3/2013		KJ439856	School of Medicine, Zhejiang University, State Key Laboratory for Diag	unknown
a/duck/zhejiang/d1-6/2013		KJ439857	School of Medicine, Zhejiang University, State Key Laboratory for Diag	unknown
a/duck/zhejiang/d1-2/2013		KJ439855	School of Medicine, Zhejiang University, State Key Laboratory for Diag	unknown
a/duck/zhejiang/d1-1/2013		KJ439854	School of Medicine, Zhejiang University, State Key Laboratory for Diag	unknown
a/duck/vietnam/lbm211/2012	EPI589352		unknown	unknown
a/duck/hunan/s1824/2012		CY146628	Harbin Veterinary Research Institute, Animal Influenza Laboratory of t	unknown
a/duck/vietnam/lbm83c-1/2012		AB847429	Avian Zoonosis Research Center,Faculty of Agriculture; 4-101 Koyama	unknown
a/muscovy_duck/vietnam/lbm115/2012	EPI596395		unknown	unknown
a/muscovy_duck/vietnam/lbm437/2013	EPI596416		unknown	unknown
a/muscovy_duck/vietnam/lbm189/2012	EPI418271		unknown	unknown
a/muscovy_duck/vietnam/lbm201/2012	EPI596401		unknown	unknown
a/duck/korea/u12/2007		JN087168	National Veterinary Research and Quarantine Service, Avian Disease	unknown
a/mallard/south_korea/n06-1355/2006	EPI1790681		unknown	unknown
a/mallard/south_korea/n07-0397/2007	EPI1790699		unknown	unknown
a/duck/korea/gj108/2007	EPI326833		unknown	unknown
a/duck/korea/gj107/2007		JN087288	National Veterinary Research and Quarantine Service, Avian Disease	unknown
a/white_peckin_duck/south_korea/p09-368/2009		MN530478	U.S. National Poultry Research Center, ARS-USDA, Exotic and Emergi	unknown
a/white_peckin_duck/south_korea/p09-373/2009		MN530481	U.S. National Poultry Research Center, ARS-USDA, Exotic and Emergi	unknown
a/duck/korea/a122/10	EPI392647		unknown	unknown
a/duck/korea/a122/2010		JN244246	Animal Plant and Fisheries Quarantine and Inspection Agency, Animal	unknown
a/white_peckin_duck/south_korea/n12-015/2012		MN530500	U.S. National Poultry Research Center, ARS-USDA, Exotic and Emergi	unknown
a/korean_native_chicken/south_korea/n12-034/2012		MN530503	U.S. National Poultry Research Center, ARS-USDA, Exotic and Emergi	unknown
a/pheasant/south_korea/n13-010/2013	EPI1790776		unknown	unknown
a/mallard/south_korea/n13-011/2013	EPI1790775		unknown	unknown
a/mallard/south_korea/n12-036/2012	EPI1790773		unknown	unknown
a/mallard/south_korea/n12-032/2012	EPI1790771		unknown	unknown
a/duck/korea/u10-2/2007		JN087160	National Veterinary Research and Quarantine Service, Avian Disease	unknown
a/duck/korea/u2-2/2007		JN087120	National Veterinary Research and Quarantine Service, Avian Disease	unknown
a/domestic_mallard/korea/hp71/2007		JN087216	National Veterinary Research and Quarantine Service, Avian Disease	unknown
a/duck/korea/hp69/2007	EPI326845		unknown	unknown
a/duck/korea/hp68/2007		JN087192	National Veterinary Research and Quarantine Service, Avian Disease	unknown
a/duck/korea/hp70/2007		JN087208	National Veterinary Research and Quarantine Service, Avian Disease	unknown
a/duck/korea/lpm86/2006		EU301237	Chungbuk National University, College of Medicine and Medical Rese	unknown
a/duck/korea/lpm09/2004		EU301223	Chungbuk National University, College of Medicine and Medical Rese	unknown
a/chicken/korea/lpm03/2004		EU301222	Chungbuk National University, College of Medicine and Medical Rese	unknown
a/duck/korea/lpm01/2004		EU301221	Chungbuk National University, College of Medicine and Medical Rese	unknown
a/chicken/korea/lpm17/2004		EU301224	Chungbuk National University, College of Medicine and Medical Rese	unknown
a/duck/korea/lpm18/2004		EU301225	Chungbuk National University, College of Medicine and Medical Rese	unknown
a/chicken/korea/lpm43/2005		EU301231	Chungbuk National University, College of Medicine and Medical Rese	unknown
a/chicken/korea/lpm44/2005		EU301232	Chungbuk National University, College of Medicine and Medical Rese	unknown
a/duck/korea/lpm23/2005		EU301227	Chungbuk National University, College of Medicine and Medical Rese	unknown
a/duck/korea/lpm22/2005		EU301226	Chungbuk National University, College of Medicine and Medical Rese	unknown
a/chicken/korea/s6/03	EPI25787		unknown	unknown

a/duck/korea/kj/2003	EPI326868		unknown	unknown
a/aquatic_bird/jeonju/kn2/2005		MW547661	Chungbuk National University, College of Medicine and Medical Rese	unknown
a/duck/korea/u2-5/2007		JN087128	National Veterinary Research and Quarantine Service, Avian Disease	unknown
a/white_peckin_duck/south_korea/n07-0182/2007		MN530417	U.S. National Poultry Research Center, ARS-USDA, Exotic and Emergi	unknown
a/duck/korea/u8-1/2007	EPI326851		unknown	unknown
a/mallard/south_korea/n07-0189/2007	EPI1790688		unknown	unknown
a/mallard/south_korea/n07-189/2007	EPI1790689		unknown	unknown
a/duck/korea/u14-1/2007		JN087176	National Veterinary Research and Quarantine Service, Avian Disease	unknown
a/duck/korea/u1-4/2007	EPI326857		unknown	unknown
a/duck/korea/dy106/2007		JN087280	National Veterinary Research and Quarantine Service, Avian Disease	unknown
a/white_peckin_duck/south_korea/n07-1621/2007		MN530444	U.S. National Poultry Research Center, ARS-USDA, Exotic and Emergi	unknown
a/white_peckin_duck/south_korea/n08-0183/2008		MN530451	U.S. National Poultry Research Center, ARS-USDA, Exotic and Emergi	unknown
a/duck/korea/gj78/2007		JN087240	National Veterinary Research and Quarantine Service, Avian Disease	unknown
a/duck/korea/gj80/2007		JN087256	National Veterinary Research and Quarantine Service, Avian Disease	unknown
a/duck/korea/gj87/2007		JN087272	National Veterinary Research and Quarantine Service, Avian Disease	unknown
a/mallard/south_korea/n07-1622/2007	EPI1790712		unknown	unknown
a/duck/korea/gj74/2007		JN087232	National Veterinary Research and Quarantine Service, Avian Disease	unknown
a/duck/korea/gj79/2007		JN087248	National Veterinary Research and Quarantine Service, Avian Disease	unknown
a/duck/korea/gj82/2007		JN087264	National Veterinary Research and Quarantine Service, Avian Disease	unknown
a/chicken/korea/lpm88/2006		EU301238	Chungbuk National University, College of Medicine and Medical Rese	unknown
a/duck/korea/u4-1/2007		JN087136	National Veterinary Research and Quarantine Service, Avian Disease	unknown
a/white_peckin_duck/south_korea/n07-0078/2007		MN530416	U.S. National Poultry Research Center, ARS-USDA, Exotic and Emergi	unknown
a/white_peckin_duck/south_korea/n07-0448/2007		MN530435	U.S. National Poultry Research Center, ARS-USDA, Exotic and Emergi	unknown
a/duck/korea/lpm36/2005		EU301228	Chungbuk National University, College of Medicine and Medical Rese	unknown
a/duck/korea/lpm38/2005		EU301229	Chungbuk National University, College of Medicine and Medical Rese	unknown
a/duck/korea/lpm39/2005		EU301230	Chungbuk National University, College of Medicine and Medical Rese	unknown
a/duck/korea/lpm91/2006		EU301239	Chungbuk National University, College of Medicine and Medical Rese	unknown
a/duck/korea/lpm92/2006		EU301240	Chungbuk National University, College of Medicine and Medical Rese	unknown
a/chicken/korea/lpm61/2005		EU301234	Chungbuk National University, College of Medicine and Medical Rese	unknown
a/duck/korea/lpm56/2005		EU301233	Chungbuk National University, College of Medicine and Medical Rese	unknown
a/chicken/korea/lpm67/2006		EU301236	Chungbuk National University, College of Medicine and Medical Rese	unknown
a/duck/korea/lpm66/2006		EU301235	Chungbuk National University, College of Medicine and Medical Rese	unknown
a/aquatic_bird/korea/jn-2/2006		EU301215	Chungbuk National University, College of Medicine and Medical Rese	unknown
a/duck/korea/js49/2004		JN087088	National Veterinary Research and Quarantine Service, Avian Disease	unknown
a/duck/korea/js53/2004	EPI326858		unknown	unknown
a/duck/korea/u14-2/2007		JN087184	National Veterinary Research and Quarantine Service, Avian Disease	unknown
a/duck/korea/jj72/2007		JN087224	National Veterinary Research and Quarantine Service, Avian Disease	unknown
a/duck/korea/u1-5/2007		JN087112	National Veterinary Research and Quarantine Service, Avian Disease	unknown
a/feline/guangdong/1/2012	EPI598219		unknown	unknown
a/feline/guangdong/1/2011		KC422461	South China Agricultural University, College of Veterinary Medicine	unknown
a/feline/korea/01/2010	EPI416234		unknown	unknown
a/feline/heilongjiang/zh/2014	EPI621136		unknown	unknown
a/feline/korea/02/2011		CY115861	KRIBB, Viral Infectious Disease Research Center	unknown
a/feline/korea/fy028/2010		KC755909	Animal, Plant and Fisheries Quarantine and Inspection Agency, Viral D	unknown
a/feline/korea/fy029/2010		KC755910	Animal, Plant and Fisheries Quarantine and Inspection Agency, Viral D	unknown
a/feline/korea/fy027/2010		KC755908	Animal, Plant and Fisheries Quarantine and Inspection Agency, Viral D	unknown
a/feline/korea/fy057/2014		KX509799	Animal and Plant Quarantine Agency, Gimcheon, Viral Disease Divisio	unknown
a/cat/indiana/m16-08618-99-1/2016	EPI2597136		unknown	unknown
a/cat/indiana/m16-08777-3-1/2016	EPI2597104		unknown	unknown
a/feline/indiana/m16-08618-100-1/2016		OQ954725	Cornell University, Baker Institute for Animal Health	unknown
a/cat/indiana/m16-08618-100-1/2016	EPI2597128		unknown	unknown

a/feline/indiana/m16-08777-3-1/2016		OQ954741	Cornell University, Baker Institute for Animal Health	unknown
a/feline/indiana/m16-08618-99-1/2016		OQ954733	Cornell University, Baker Institute for Animal Health	unknown
a/feline/indiana/m16-09000-4-1/2016		OQ954749	Cornell University, Baker Institute for Animal Health	unknown
a/cat/indiana/m16-09000-4-1/2016	EPI2597112		unknown	unknown
a/dog/indiana/m16-09378-32-1/2016	EPI2597120		unknown	unknown
a/equus_caballus/usa/149632/2018	EPI1526366		unknown	unknown
a/equus_caballus/usa/154390/2018	EPI1526390		unknown	unknown
a/felis_catus/usa/047732/2018	EPI1567172		unknown	unknown
a/canis_lupus_familiaris/usa/218592/2017	EPI1526438		unknown	unknown
a/canis_lupus_familiaris/usa/150620/2018	EPI1526374		unknown	unknown
a/canis_lupus_familiaris/usa/152386/2018	EPI1526382		unknown	unknown
a/canis_lupus_familiaris/usa/007781/2018	EPI1526358		unknown	unknown
a/canis_lupus_familiaris/usa/006974/2018	EPI1526342		unknown	unknown
a/canis_lupus_familiaris/usa/007780/2018	EPI1526350		unknown	unknown
a/canis_lupus_familiaris/usa/000915/2018	EPI1526334		unknown	unknown
a/canis_lupus_familiaris/usa/188226/2017	EPI1526414		unknown	unknown
a/canis_lupus_familiaris/usa/118191/2017	EPI1526398		unknown	unknown
a/canis_lupus_familiaris/usa/188297/2017	EPI1526422		unknown	unknown
a/chicken/shantou/175/2022		OQ292749	The University of Hong Kong, School of Public Health	unknown
a/chicken/shantou/4427/2021		OQ292829	The University of Hong Kong, School of Public Health	unknown
a/silkie_chicken/shantou/4771/2021		OQ293733	The University of Hong Kong, School of Public Health	unknown
a/chicken/shantou/4424/2021		OQ292813	The University of Hong Kong, School of Public Health	unknown
a/chicken/guangzhou/199/2022		OQ291909	The University of Hong Kong, School of Public Health	unknown
a/chicken/huizhou/175/2022		OQ292021	The University of Hong Kong, School of Public Health	unknown
a/silkie_chicken/dongguan/781/2022		OQ293477	The University of Hong Kong, School of Public Health	unknown
a/chicken/jiangsu/c312/2022	EPI2047645		China Agricultural University	China Agricultural University
a/chicken/guangdong/f1201/2021	EPI2047593		China Agricultural University	China Agricultural University
a/silkie_chicken/shantou/6419/2021		OQ293781	The University of Hong Kong, School of Public Health	unknown
a/chicken/guangdong/778/2022	EPI2595281		unknown	unknown
a/chicken/guangdong/6799/2021	EPI2593751		unknown	unknown
a/chicken/huizhou/17/2022		OQ292013	The University of Hong Kong, School of Public Health	unknown
a/chicken/guangdong/17/2022	EPI2594525		unknown	unknown
a/chicken/guangdong/4683/2021	EPI2595166		unknown	unknown
a/chicken/shantou/5171/2021		OQ292989	The University of Hong Kong, School of Public Health	unknown
a/chicken/guangdong/6665/2021	EPI2594291		unknown	unknown
a/chicken/guangdong/2550/2021	EPI2594303		unknown	unknown
a/chicken/shantou/6665/2021		OQ293253	The University of Hong Kong, School of Public Health	unknown
a/chicken/jiangxi/38623/2021	EPI2595367		unknown	unknown
a/chicken/dongguan/457/2022		OQ291781	The University of Hong Kong, School of Public Health	unknown
a/chicken/shantou/5811/2021		OQ293053	The University of Hong Kong, School of Public Health	unknown
a/chicken/jiangxi/10580/2022	EPI2595260		unknown	unknown
a/chicken/guangdong/4689/2021	EPI2595142		unknown	unknown
a/chicken/guangdong/6771/2021	EPI2593799		unknown	unknown
a/chicken/shantou/6745/2021		OQ293325	The University of Hong Kong, School of Public Health	unknown
a/silkie_chicken/shantou/495/2022		OQ293757	The University of Hong Kong, School of Public Health	unknown
a/chicken/guangdong/971/2022	EPI2594737		unknown	unknown
a/silkie_chicken/huizhou/114/2022		OQ293501	The University of Hong Kong, School of Public Health	unknown
a/chicken/shantou/475/2022		OQ292941	The University of Hong Kong, School of Public Health	unknown
a/chicken/guangdong/333/2022	EPI2595115		unknown	unknown
a/chicken/guangdong/5669/2021	EPI2593869		unknown	unknown
a/chicken/guangdong/2568/2021	EPI2594279		unknown	unknown

a/chicken/guangdong/876/2022	EPI2595209		unknown	unknown
a/chicken/guangdong/6717/2021	EPI2594171		unknown	unknown
a/chicken/shantou/6673/2021		OQ293261	The University of Hong Kong, School of Public Health	unknown
a/chicken/huizhou/456/2022		OQ292197	The University of Hong Kong, School of Public Health	unknown
a/chicken/dongguan/397/2022		OQ291701	The University of Hong Kong, School of Public Health	unknown
a/chicken/guangdong/6650/2021	EPI2594573		unknown	unknown
a/chicken/huizhou/305/2022		OQ292133	The University of Hong Kong, School of Public Health	unknown
a/chicken/shantou/6129/2021		OQ293109	The University of Hong Kong, School of Public Health	unknown
a/chicken/guangdong/6313/2021	EPI2594267		unknown	unknown
a/chicken/guangdong/5171/2021	EPI2593922		unknown	unknown
a/chicken/guangzhou/4525/2021		OQ291949	The University of Hong Kong, School of Public Health	unknown
a/chicken/huizhou/216/2022		OQ292045	The University of Hong Kong, School of Public Health	unknown
a/chicken/guangdong/6772/2021	EPI2594067		unknown	unknown
a/chicken/shantou/6772/2021		OQ293341	The University of Hong Kong, School of Public Health	unknown
a/chicken/dongguan/450/2022		OQ291773	The University of Hong Kong, School of Public Health	unknown
a/chicken/dongguan/3707/2021		OQ291677	The University of Hong Kong, School of Public Health	unknown
a/chicken/guangdong/364/2022	EPI2594703		unknown	unknown
a/chicken/dongguan/426/2022		OQ291757	The University of Hong Kong, School of Public Health	unknown
a/chicken/henan/f0316/2022	EPI2047318		China Agricultural University	China Agricultural University
a/chicken/jiangxi/11767/2022	EPI2595871		unknown	unknown
a/chicken/shantou/6785/2021		OQ293357	The University of Hong Kong, School of Public Health	unknown
a/chicken/jiangxi/8560/2022	EPI2595617		unknown	unknown
a/chicken/shantou/6783/2021		OQ293349	The University of Hong Kong, School of Public Health	unknown
a/chicken/jiangxi/8068/2022	EPI2595732		unknown	unknown
a/chicken/guangdong/4623/2021	EPI2593983		unknown	unknown
a/chicken/guangdong/6145/2021	EPI2593861		unknown	unknown
a/chicken/shantou/5669/2021		OQ293045	The University of Hong Kong, School of Public Health	unknown
a/silkie_chicken/shantou/6433/2021		OQ293789	The University of Hong Kong, School of Public Health	unknown
a/chicken/guangdong/168/2022	EPI2594557		unknown	unknown
a/chicken/guangdong/6637/2021	EPI2594339		unknown	unknown
a/chicken/jiangxi/12248/2022	EPI2595791		unknown	unknown
a/silkie_chicken/huizhou/2615/2021		OQ293533	The University of Hong Kong, School of Public Health	unknown
a/chicken/dongguan/40/2022		OQ291709	The University of Hong Kong, School of Public Health	unknown
a/chicken/jiangxi/12291/2022	EPI2595753		unknown	unknown
a/chicken/guangdong/350/2022	EPI2594450		unknown	unknown
a/chicken/shantou/1715/2022		OQ292733	The University of Hong Kong, School of Public Health	unknown
a/chicken/jiangxi/11741/2022	EPI2595470		unknown	unknown
a/chicken/shantou/52/2022		OQ293005	The University of Hong Kong, School of Public Health	unknown
a/chicken/guangdong/1/2022	EPI2594652		unknown	unknown
a/silkie_chicken/huizhou/411/2022		OQ293565	The University of Hong Kong, School of Public Health	unknown
a/chicken/shantou/4689/2021		OQ292901	The University of Hong Kong, School of Public Health	unknown
a/chicken/guangdong/6325/2021	EPI2594051		unknown	unknown
a/chicken/guangdong/6803/2021	EPI2594605		unknown	unknown
a/chicken/shantou/41/2022		OQ292773	The University of Hong Kong, School of Public Health	unknown
a/chicken/dongguan/93/2022		OQ291885	The University of Hong Kong, School of Public Health	unknown
a/chicken/shantou/6395/2021		OQ293173	The University of Hong Kong, School of Public Health	unknown
a/silkie_chicken/shantou/4323/2021		OQ293637	The University of Hong Kong, School of Public Health	unknown
a/chicken/shantou/4677/2021		OQ292885	The University of Hong Kong, School of Public Health	unknown
a/chicken/guangdong/6745/2021	EPI2594083		unknown	unknown
a/chicken/guangdong/684/2022	EPI2594876		unknown	unknown
a/chicken/guangdong/356/2022	EPI2595438		unknown	unknown

a/chicken/guangdong/489/2022	EPI2593954		unknown	unknown
a/chicken/huizhou/463/2022		OQ292205	The University of Hong Kong, School of Public Health	unknown
a/chicken/dongguan/246/2022		OQ291645	The University of Hong Kong, School of Public Health	unknown
a/chicken/guangzhou/1329/2022		OQ291893	The University of Hong Kong, School of Public Health	unknown
a/chicken/huizhou/361/2022		OQ292189	The University of Hong Kong, School of Public Health	unknown
a/chicken/jiangxi/8047/2022	EPI2595770		unknown	unknown
a/chicken/guangdong/93/2022	EPI2595113		unknown	unknown
a/chicken/huizhou/209/2022		OQ292037	The University of Hong Kong, School of Public Health	unknown
a/silkie_chicken/huizhou/2750/2021		OQ293557	The University of Hong Kong, School of Public Health	unknown
a/chicken/dongguan/411/2022		OQ291733	The University of Hong Kong, School of Public Health	unknown
a/chicken/dongguan/888/2022		OQ291869	The University of Hong Kong, School of Public Health	unknown
a/chicken/guangdong/121/2022	EPI2594786		unknown	unknown
a/chicken/huizhou/12/2022		OQ291997	The University of Hong Kong, School of Public Health	unknown
a/chicken/guangdong/6383/2021	EPI2594541		unknown	unknown
a/chicken/guangdong/5632/2021	EPI2594002		unknown	unknown
a/chicken/china/guangdong_01/2022		ON626399	South China Agricultural University, Animal Science	unknown
a/silkie_chicken/shantou/343/2022		OQ293581	The University of Hong Kong, School of Public Health	unknown
a/chicken/guangdong/493/2022	EPI2594637		unknown	unknown
a/chicken/guangdong/104/2022	EPI2594621		unknown	unknown
a/chicken/huizhou/190/2022		OQ292029	The University of Hong Kong, School of Public Health	unknown
a/chicken/jiangxi/6499/2022	EPI2595303		unknown	unknown
a/chicken/guangdong/4421/2021	EPI2595506		unknown	unknown
a/chicken/guangdong/246/2022	EPI2594751		unknown	unknown
a/chicken/huizhou/320/2022		OQ292149	The University of Hong Kong, School of Public Health	unknown
a/chicken/guangdong/001/2022	EPI2595572		unknown	unknown
a/chicken/jiangxi/10526/2022	EPI2594852		unknown	unknown
a/chicken/shantou/4501/2021		OQ292853	The University of Hong Kong, School of Public Health	unknown
a/chicken/guangdong/6737/2021	EPI2594099		unknown	unknown
a/chicken/guangdong/6735/2021	EPI2594115		unknown	unknown
a/chicken/jiangxi/740/2022	EPI2594968		unknown	unknown
a/chicken/guangdong/1329/2022	EPI2595089		unknown	unknown
a/chicken/guangdong/173/2022	EPI2595674		unknown	unknown
a/chicken/guangdong/4463/2021	EPI2595315		unknown	unknown
a/chicken/guangdong/85/2022	EPI2594670		unknown	unknown
a/chicken/jiangxi/3758/2022	EPI2595591		unknown	unknown
a/chicken/jiangxi/8076/2022	EPI2595713		unknown	unknown
a/chicken/guangzhou/856/2022		OQ291957	The University of Hong Kong, School of Public Health	unknown
a/chicken/guangdong/6521/2021	EPI2594435		unknown	unknown
a/chicken/guangdong/1369/2022	EPI2595057		unknown	unknown
a/chicken/guangdong/856/2022	EPI2595284		unknown	unknown
a/chicken/guangdong/567/2022	EPI2594934		unknown	unknown
a/chicken/guangdong/4629/2021	EPI2594031		unknown	unknown
a/chicken/guangdong/416/2022	EPI2595412		unknown	unknown
a/chicken/guangdong/6105/2021	EPI2593753		unknown	unknown
a/chicken/jiangxi/3711/2022	EPI2595463		unknown	unknown
a/chicken/shantou/6134/2021		OQ293125	The University of Hong Kong, School of Public Health	unknown
a/chicken/guangdong/916/2022	EPI2595137		unknown	unknown
a/chicken/guangdong/745/2022	EPI2594354		unknown	unknown
a/chicken/jiangxi/6991/2022	EPI2595602		unknown	unknown
a/silkie_chicken/shantou/6534/2021		OQ293813	The University of Hong Kong, School of Public Health	unknown
a/chicken/guangdong/196/2022	EPI2595064		unknown	unknown

a/chicken/shantou/1709/2022		OQ292725	The University of Hong Kong, School of Public Health	unknown
a/chicken/shantou/6637/2021		OQ293229	The University of Hong Kong, School of Public Health	unknown
a/chicken/shantou/479/2022		OQ292949	The University of Hong Kong, School of Public Health	unknown
a/chicken/shantou/6133/2021		OQ293117	The University of Hong Kong, School of Public Health	unknown
a/chicken/dongguan/879/2022		OQ291861	The University of Hong Kong, School of Public Health	unknown
a/silkie_chicken/huizhou/121/2022		OQ293509	The University of Hong Kong, School of Public Health	unknown
a/chicken/jiangxi/11803/2022	EPI2595831		unknown	unknown
a/silkie_chicken/shantou/6531/2021		OQ293797	The University of Hong Kong, School of Public Health	unknown
a/chicken/fujian/f0215/2022	EPI2167530		China Agricultural University	China Agricultural University
a/chicken/guangdong/3506/2021	EPI2594626		unknown	unknown
a/chicken/guangdong/4621/2021	EPI2593967		unknown	unknown
a/chicken/guangdong/297/2022	EPI2594780		unknown	unknown
a/chicken/shantou/4600/2021		OQ292877	The University of Hong Kong, School of Public Health	unknown
a/chicken/anhui/fd3/2022	EPI2047351		China Agricultural University	China Agricultural University
a/chicken/guangdong/842/2022	EPI2595257		unknown	unknown
a/silkie_chicken/shantou/350/2022		OQ293613	The University of Hong Kong, School of Public Health	unknown
a/chicken/shantou/1/2022		OQ292709	The University of Hong Kong, School of Public Health	unknown
a/chicken/huizhou/2601/2021		OQ292101	The University of Hong Kong, School of Public Health	unknown
a/chicken/jiangxi/1975/2022	EPI2595677		unknown	unknown
a/chicken/guangdong/6104/2021	EPI2593769		unknown	unknown
a/silkie_chicken/huizhou/2626/2021		OQ293541	The University of Hong Kong, School of Public Health	unknown
a/chicken/shantou/6313/2021		OQ293149	The University of Hong Kong, School of Public Health	unknown
a/chicken/guangzhou/4398/2021		OQ291933	The University of Hong Kong, School of Public Health	unknown
a/chicken/dongguan/416/2022		OQ291741	The University of Hong Kong, School of Public Health	unknown
a/chicken/guangdong/6419/2021	EPI2594186		unknown	unknown
a/chicken/jiangxi/11782/2022	EPI2595847		unknown	unknown
a/chicken/jiangxi/11775/2022	EPI2595855		unknown	unknown
a/chicken/guangdong/111/2022	EPI2594563		unknown	unknown
a/silkie_chicken/huizhou/267/2022		OQ293549	The University of Hong Kong, School of Public Health	unknown
a/chicken/guangdong/4525/2021	EPI2595161		unknown	unknown
a/chicken/shantou/4693/2021		OQ292917	The University of Hong Kong, School of Public Health	unknown
a/chicken/shantou/6720/2021		OQ293301	The University of Hong Kong, School of Public Health	unknown
a/chicken/guangdong/4422/2021	EPI2595477		unknown	unknown
a/chicken/shantou/6737/2021		OQ293317	The University of Hong Kong, School of Public Health	unknown
a/chicken/shantou/6523/2021		OQ293205	The University of Hong Kong, School of Public Health	unknown
a/chicken/shantou/6104/2021		OQ293093	The University of Hong Kong, School of Public Health	unknown
a/chicken/guangdong/325/2022	EPI2595139		unknown	unknown
a/chicken/guangdong/5500/2021	EPI2593877		unknown	unknown
a/chicken/guangdong/463/2022	EPI2595236		unknown	unknown
a/chicken/guangdong/401/2022	EPI2595476		unknown	unknown
a/chicken/guangdong/4699/2021	EPI2594255		unknown	unknown
a/chicken/dongguan/375/2022		OQ291685	The University of Hong Kong, School of Public Health	unknown
a/chicken/huizhou/684/2022		OQ292245	The University of Hong Kong, School of Public Health	unknown
a/chicken/dongguan/916/2022		OQ291877	The University of Hong Kong, School of Public Health	unknown
a/chicken/dongguan/3758/2021		OQ291693	The University of Hong Kong, School of Public Health	unknown
a/chicken/guangdong/f0103/2022	EPI2047426		China Agricultural University	China Agricultural University
a/chicken/guangdong/6523/2021	EPI2594411		unknown	unknown
a/chicken/shantou/191/2022		OQ292757	The University of Hong Kong, School of Public Health	unknown
a/chicken/shantou/6719/2021		OQ293293	The University of Hong Kong, School of Public Health	unknown
a/silkie_chicken/shantou/4609/2021		OQ293653	The University of Hong Kong, School of Public Health	unknown
a/chicken/huizhou/297/2022		OQ292125	The University of Hong Kong, School of Public Health	unknown

a/silkie_chicken/shantou/745/2022		OQ293837	The University of Hong Kong, School of Public Health	unknown
a/chicken/guangdong/4599/2021	EPI2595238		unknown	unknown
a/chicken/huizhou/325/2022		OQ292157	The University of Hong Kong, School of Public Health	unknown
a/chicken/guangdong/4630/2021	EPI2594047		unknown	unknown
a/chicken/guangdong/190/2022	EPI2594495		unknown	unknown
a/chicken/guangdong/475/2022	EPI2594207		unknown	unknown
a/chicken/huizhou/487/2022		OQ292213	The University of Hong Kong, School of Public Health	unknown
a/chicken/guangdong/734/2022	EPI2595310		unknown	unknown
a/chicken/guangdong/349/2022	EPI2594159		unknown	unknown
a/chicken/shantou/6145/2021		OQ293133	The University of Hong Kong, School of Public Health	unknown
a/chicken/guangdong/4321/2021	EPI2594426		unknown	unknown
a/silkie_chicken/shantou/4774/2021		OQ293741	The University of Hong Kong, School of Public Health	unknown
a/silkie_chicken/dongguan/495/2022		OQ293469	The University of Hong Kong, School of Public Health	unknown
a/chicken/guangdong/5386/2021	EPI2593893		unknown	unknown
a/chicken/shantou/4691/2021		OQ292909	The University of Hong Kong, School of Public Health	unknown
a/silkie_chicken/dongguan/3829/2021		OQ293461	The University of Hong Kong, School of Public Health	unknown
a/chicken/guangdong/6720/2021	EPI2594131		unknown	unknown
a/silkie_chicken/shantou/4629/2021		OQ293701	The University of Hong Kong, School of Public Health	unknown
a/chicken/guangdong/4367/2021	EPI2595530		unknown	unknown
a/chicken/guangdong/2615/2021	EPI2594858		unknown	unknown
a/chicken/shantou/6147/2021		OQ293141	The University of Hong Kong, School of Public Health	unknown
a/chicken/jiangxi/3269/2022	EPI2595895		unknown	unknown
a/silkie_chicken/shantou/4321/2021		OQ293629	The University of Hong Kong, School of Public Health	unknown
a/chicken/guangdong/5181/2021	EPI2593909		unknown	unknown
a/chicken/guangzhou/4313/2021		OQ291917	The University of Hong Kong, School of Public Health	unknown
a/chicken/shantou/4421/2021		OQ292797	The University of Hong Kong, School of Public Health	unknown
a/chicken/shantou/5500/2021		OQ293029	The University of Hong Kong, School of Public Health	unknown
a/chicken/shantou/6803/2021		OQ293389	The University of Hong Kong, School of Public Health	unknown
a/chicken/guangdong/f0210/2022	EPI2047439		China Agricultural University	China Agricultural University
a/chicken/guangdong/6147/2021	EPI2594034		unknown	unknown
a/chicken/guangdong/879/2022	EPI2595374		unknown	unknown
a/chicken/huizhou/231/2022		OQ292061	The University of Hong Kong, School of Public Health	unknown
a/silkie_chicken/dongguan/790/2022		OQ293485	The University of Hong Kong, School of Public Health	unknown
a/chicken/jiangxi/12309/2022	EPI2595696		unknown	unknown
a/chicken/guangdong/2542/2021	EPI2594327		unknown	unknown
a/silkie_chicken/dongguan/3506/2021		OQ293453	The University of Hong Kong, School of Public Health	unknown
a/chicken/guangdong/42/2022	EPI2595554		unknown	unknown
a/chicken/guangdong/426/2022	EPI2595346		unknown	unknown
a/chicken/guangdong/f0314/2022	EPI2047571		China Agricultural University	China Agricultural University
a/chicken/guangdong/466/2022	EPI2594896		unknown	unknown
a/chicken/guangdong/898/2022	EPI2594966		unknown	unknown
a/chicken/guangdong/3758/2021	EPI2594577		unknown	unknown
a/chicken/guangdong/6713/2021	EPI2594195		unknown	unknown
a/chicken/guangdong/4428/2021	EPI2595349		unknown	unknown
a/chicken/anhui/ckah01/2022		OP024201	Yangzhou University, College of Veterinary Medicine	unknown
a/chicken/guangdong/4600/2021	EPI2595214		unknown	unknown
a/chicken/guangdong/4609/2021	EPI2593935		unknown	unknown
a/chicken/huizhou/50/2022		OQ292221	The University of Hong Kong, School of Public Health	unknown
a/chicken/dongguan/50/2022		OQ291797	The University of Hong Kong, School of Public Health	unknown
a/chicken/shantou/196/2022		OQ292765	The University of Hong Kong, School of Public Health	unknown
a/chicken/guangdong/4450/2021	EPI2595317		unknown	unknown

a/chicken/zhejiang/ckzj04/2022		OP024225	Yangzhou University, College of Veterinary Medicine	unknown
a/chicken/guangdong/4425/2021	EPI2595413		unknown	unknown
a/chicken/guangdong/4501/2021	EPI2595285		unknown	unknown
a/chicken/jiangxi/12303/2022	EPI2595715		unknown	unknown
a/chicken/guangdong/6535/2021	EPI2594306		unknown	unknown
a/chicken/guangdong/305/2022	EPI2595212		unknown	unknown
a/chicken/guangdong/24/2022	EPI2594775		unknown	unknown
a/chicken/huizhou/310/2022		OQ292141	The University of Hong Kong, School of Public Health	unknown
a/chicken/jiangxi/12277/2022	EPI2595772		unknown	unknown
a/chicken/guangdong/6103/2021	EPI2593785		unknown	unknown
a/chicken/guangdong/320/2022	EPI2595163		unknown	unknown
a/chicken/guangdong/4755/2021	EPI2594079		unknown	unknown
a/chicken/jiangsu/ckjs03/2022		OP024217	Yangzhou University, College of Veterinary Medicine	unknown
