# Supplementary material for: Fatal human H3N8 influenza virus has a moderate pandemic risk
Source: PLoS Pathog. 2026 Mar 3;22(3):e1013586. doi: 10.1371/journal.ppat.1013586 (PMC12970972; doi:10.1371/journal.ppat.1013586)
Supplement: Attachment — Submitted filename: 260118_H3N8_PlosPath_Response resub.docx [file ppat.1013586.s005.docx]

**Response to reviewers**

PPATHOGENS-D-25-02436
Fatal Human H3N8 Influenza Virus has a Moderate Pandemic Risk
PLOS Pathogens

Reviewer’s comments and questions will appear in black text, *responses will appear in italic blue text*.

**Reviewers' Comments:**

**Part I - Summary**
Please use this section to discuss strengths/weaknesses of study, novelty/significance, general execution and scholarship.

Reviewer #1: This manuscript by Le Sage and colleagues evaluates the pandemic potential of a low path again H3N8 strain which has made several dead end jumps into humans, causing disease and death. They run through a battery of assays which demonstrate that this virus may be poised for emergence. Overall the manuscript is well written and I believe the results will be of broad interest to the field. More context as to why the authors chose to compare H3N8 to pH1N1 would be helpful. For the antibody history comparison this is ostensibly to show that pH1N1 was able to emerge even in spite of the presence of sera reactivity. It might be intuitive to a lot of readers to make the comparison to seasonal H3 viruses. Additionally, given that there is at least 1 study on H3N8 pandemic potential it would help to highlight what is known and unknown earlier in the paper to put the experiments into better context.

*Response: We thank the reviewer for their comments and recognizing the importance to the field. We have included contemporary H3N2 viruses for comparison when appropriate, see revised figures 2 and 3. In addition, lines 136-138 and 147-148 have been added to provide more context. We have also adjusted the text to emphasize the novelty of our current study which includes a comprehensive analysis of the human lethal strain of H3N8 and utilization of pre-immune ferrets to define the transmission fitness of this circulating subclade.*

Reviewer #2: The authors reconstituted an avian H3N8 virus using reverse genetics that caused a fatal human infection to investigate its pandemic potentials characterized by replication ability in human bronchial epithelial cells (HBEC), sialic acid receptor binding preference (2,3-linked or 2,6-linked), airborne and direct-contact transmission in pre-immune ferrets and pre-existing antibody responses in humans to it. Despite of lack of cross-neutralizing antibodies in human population, efficient replication of virus in HBEC, dual receptor binding, similar decay rate to pandemic H1N1, the virus was transmitted via direct-contact, but not airborne route in ferrets with immunity to the historical H3N2 virus lower its pandemic potentials to moderate. The study is well conducted with a few comments to be addressed.

*Response: We thank the reviewer for their comments and their concerns are addressed below.*

**Part II – Major Issues: Key Experiments Required for Acceptance**
Please use this section to detail the key new experiments or modifications of existing experiments that should be absolutely required to validate study conclusions.

Reviewer #1: 1. Understanding if H3N8 can transmit in the face of preexisting H3N2 immunity (which most humans above 2-3yo will have) is critical. However the negative result is difficult to interpret without a positive control. Does this virus transmit to naive animals by aerosol? In the absence of these data the authors should temper their conclusions and acknowledge this limitation.

*Response: We thank the reviewer for this perspective and have tempered our conclusions to suggest that this H3N8 virus may not transmit through the air and not that prior immunity is a barrier to this feature. We consider prior immunity to be critical feature in the assessment of transmissibility in the human population thus believe that transmission in immunologically naïve ferrets will be misleading and overstate the possible pandemic threat. Please see revised text lines 183-184 (subsection heading); 186-188; 209; 238-241 and title of figure 5.*

2. It would be helpful to have a basis of comparison for Fig 3 C and D (even if just historical data). For example are these binding levels similar to seasonal flu viruses? Lower?

*Response: Thank you for the suggestion and we agree that a point of comparison is important. We have included binding profiles for the human seasonal H3N2 virus A/Darwin/6/2021 H3N2 virus. Please see the revised Figure 3.*

Reviewer #2: NA

**Part III – Minor Issues: Editorial and Data Presentation Modifications**
Please use this section for editorial suggestions as well as relatively minor modifications of existing data that would enhance clarity.

Reviewer #1: 1. Methods state serum were used in fig 1, should this be fig 2?
*Response: We thank the reviewer for pointing this out and have corrected it.*

2. Results section and methods discuss serum from 2020 but these data do not appear to be actually shown in the figure
*Response: The serum samples analyzed in Figure 2 A were collected from individuals in 2020 with the specified birth years, but not from individuals with the birth year of 2020, which is why 2020 does not appear on the graph.*

3. Stats for 4B, D should be shown
*Response: We appreciate the suggestion and have performed some statistical analysis as well as adjusted the text related describing the results (see lines 153-157 and 173-175). A t-test of the pH of inactivation was significantly different, unsurprisingly, and we describe this in the figure legend. Statistical analysis for figure 4D is more challenging, since an EC50 is difficult to calculate because the curves are consistently S-shaped. Therefore, we have modified the conclusion and provided the rationale for assuming that this phenotype of NA activity would satisfy the feature described in the triage framework (see lines 173-175).*

4. The methods rescue section mentions MP several times which should be corrected to NP

*Response: We thank the reviewer for pointing out these typos and have corrected them.*

5. Fig 1 was low resolution and difficult to read.

*Response: We have included a higher resolution Figure 1 file.*

Reviewer #2: -Please improve the quality of figure 1

*Response: We have included a higher resolution Figure 1 file.*

-Please add line number and page number

*Response: We have added these.*

-Figure 4C, please correct the black and red symbols of dots to bars, which more accurately represent the groups.

*Response: We thank the reviewer for this comment and have adjusted the figure such that each HBE ASL is a separate bar and the technical replicates are presented as individual points within each bar. Please see revised Figure 4C.*

-Please clarify if the experimental stock of the H3N8 virus was sequence-confirmed?

*Response: The plasmids used to synthesize the virus stock were sequence confirmed, but the stock itself was not sequence confirmed prior to the experiments. This has been added to the MM (line 347)*

-Please provide more details on the glycans used in glycan ELISAs, how were they obtained (e.g, from commercial kit or made in-house)

*Response: The MM has been modified to include this information and the reference for the glycan ELISA. Please see lines:384*

-How did the authors validate the A/Perth/09 H3N2 immunity is waning in ferrets?

*Response: We appreciate this statement in the MM may have been confusing, and have clarified it, see line 449-450.*

-The lack of airborne transmission is found in ferret immunized with H3N2 from 2009. What are the expectations for airborne transmission in naïve individuals or individuals born after 2009? Please discuss.

*Response: This is a very important point, and we thank the reviewer for raising it. We have added a paragraph in the discussion on this topic (lines 246-254). It is hard to predict how prior immunity from multiple seasonal exposures will differ from the single imprint induced in our study, and whether the impact of more recent seasonal viruses will produce a different outcome. In addition, we added text, based on Reviewer 1’s suggestion, regarding immunologically naïve ferrets in discussion (lines 238-241).*

- In the discussion, shouldn’t the statement “It is important to note that in the 1918 and 2019 H1N1 human pandemics some early isolates exhibited dual receptor specificity resulting from a single mutation from the avian virus progenitor (39-41).” Refer to 2009 H1N1 instead of 2019 H1N1?

*Response: We thank the reviewer for point out this typo and have corrected it.*

-Please described the biosafety levels in which the H3N8 virus was handled since it has a fatal outcome in humans, or justify the use of lower containment conditions

*Response: Based on the approved IACUC and IBC applications at the University of Pittsburgh, low pathogenic avian influenza strains are handled at BSL2 with enhanced conditions. This includes PPE such as N95s, Tyvek, lab coat, double gloves, hair bonnet, double shoe covers, and face shield during handling of ferrets. For in vitro work this includes using an enclosed space, disposable lab coat, safety glasses, and N95.*
